# The nonstructural protein 1 of respiratory syncytial virus hijacks host mitophagy as a novel mitophagy receptor to evade the type I IFN response in HEp-2 cells

**DOI:** 10.1128/mbio.01480-23

**Published:** 2023-11-01

**Authors:** Jing Cheng, Yutong Wang, Lizheng Yin, Wenzhang Liang, Jing Zhang, Cuiqing Ma, Yu Zhang, Bo Liu, Jiachao Wang, Weiting Zhao, Miao Li, Lin Wei

**Affiliations:** 1 Department of Immunology, Hebei Medical University, Shijiazhuang, Hebei, China; 2 Key Laboratory of Immune Mechanism and Intervention on Serious Disease in Hebei Province, Shijiazhuang, Hebei, China; 3 Department of Pathogen Biology, Hebei Medical University, Shijiazhuang, Hebei, China; Washington University in St. Louis, St. Louis, Missouri, USA; Washington University School of Medicine, St. Louis, Missouri, USA

**Keywords:** RSV NS1 protein, mitophagy, IFNβ, TUFM, viral replication

## Abstract

Mitochondria are good targets for viruses to manipulate their hosts. However, it remains obscure whether respiratory syncytial virus (RSV) target mitochondria to suppress the type I interferon (IFN) responses. Here, we show that nonstructural protein 1 (NS1) protein of RSV interacts with Tu translation elongation factor mitochondrial (TUFM), which can lead to its localization in mitochondria and finally induce TUFM-dependent mitophagy and inhibition of IFNβ. Mechanically, NS1-mediated TUFM-dependent mitophagy does not depend on the PINK1-PARKIN pathway and classic mitophagy receptors. Importantly, NS1 may act as a new receptor protein to bridge mitochondria and autophagosomes by interacting with TUFM and LC3B. The LIR motif of NS1 protein is essential for its interaction with LC3B and is of great importance for its mitophagy induction and IFNβ suppression. Finally, NS1-induced TUFM-dependent mitophagy was essential for its attenuated IFNβ response using autophagy-deficient cells and mice. Our study provides a novel mitophagy receptor molecular and a new antiviral option by suppressing antiviral innate immune via targeting TUFM-dependent mitophagy.

**IMPORTANCE:**

It is a worthy concern for us to understand virus-host interactions which affect progression and prognosis of disease. We demonstrated that the non-structural protein 1 of respiratory syncytial virus (RSV NS1) may act as a novel mitophagy receptor to induce mitophagy by binding LC3B and mitochondrial protein TUFM, and finally dampen interferon (IFN) responses induced by RIG1 and RSV infection. TUFM is beneficial for RSV replication *in vivo* and *vitro*. It is new and interesting that RSV NS1 may function as a mitophagy receptor to interact with LC3B. The LIR motif of NS1 protein is essential for its interaction with LC3B. We further confirm that RSV NS1 inhibited IFNβ response and promoted RSV replication in autophagy-dependent mechanisms *in vivo* and *vitro*. Our study contributes to understanding virus-host interaction, enriching our insights into RSV pathogenic mechanism and exploiting new antiviral treatments targeting TUFM.

## INTRODUCTION

After the COVID-19 pandemic, a worrying surge in RSV infection has been reported in some countries. RSV is an enveloped virus with a negative-stranded RNA genome, which is the leading cause of acute lower respiratory tract infection (ALRI) in infants and the elderly worldwide (1). In 2015, there were 33.1 million episodes of RSV-ALRI in children younger than 5 years, which resulted in about 3.2 million hospital admissions and 60 thousand in-hospital deaths (2). Although RSV has caused a great disease burden, effective prophylactic and treatment options are unavailable. Lack of understanding of virus-host interactions consequently restricts our ability to identify attractive anti-RSV drug targets and develop novel therapeutics.

Upon RSV infection, PRRs recognize RSV-derived PAMP and then activate downstream signaling cascades leading to the production of type I and III interferons (IFNs) and proinflammatory cytokines (3). Type I IFNs are critical for combating virus infection by mounting an antiviral state in virus-infected and neighboring cells. However, accumulated clinical studies have shown that RSV is a poor inducer of type I IFN, closely related to disease severity (4–6). RSV has evolved mechanisms to subvert host interferon responses, mostly notably by RSV non-structural protein NS1 and NS2 (7). Of the two non-structural proteins, NS1 appears to act as a more potent and dominant suppressor of IFN signaling (8). It has been proposed that a “degradosome” complex composed of RSV-NS1 and several unknown host factors was formed in mitochondria (9). Besides, as stated previously, RSV-NS1 interacted with MAVS (10). All these studies hint at the possibility of mitochondrial localization of RSV-NS1. Mitochondria are highly dynamic organelles that continuously undergo fusion and fission. These delicately regulated processes, namely mitochondria dynamics, are critical for maintaining cellular homeostasis and bioenergetics (11). Moreover, mitochondria serve as an IFN-signaling platform to activate RIG1 or MDA5 pathway by MAVS (12–14). Unsurprisingly, many viruses target mitochondria to manipulate mitochondrial functions and host immunity for their propagation. Japanese encephalitis virus (JEV) NS4A protein localizes to mitochondria and interacts with PTEN-induced kinase 1 (PINK1) to promote mitochondrial fission and mitophagy and favor JEV replication (15). Rotaviruses (RVs) VP3 localizes to the mitochondria and blocks IFNλ production in RV-infected intestinal epithelial cells through mediating phosphorylation and subsequent proteasomal degradation of MAVS (16). HCV NS3A/4A protein evades IFNβ response by colocalizing with and cleaving MAVS (17). However, it is still unclear whether RSV-NS1 localizes to mitochondria and affects mitochondria homeostasis. If yes, further research is needed to conﬁrm the relationship between this effect of RSV-NS1 and its inhibitory role in IFN signaling.

Mitochondria dynamics can be directly affected by viral proteins targeting mitochondria, which is a prerequisite for mitophagy. Mitophagy, a specific type of macro-autophagy, is an evolutionarily conserved cellular process that can selectively remove dysfunctional or excessive mitochondria via specific engulfment of mitochondria for subsequent lysosome degradation (18). Virus-induced mitophagy has been demonstrated to attenuate interferon responses. Severe acute respiratory syndrome coronavirus 2 (SARS-CoV-2) ORF10 inhibits the IFN-I signaling pathway by inducing mitophagy-mediated MAVS degradation (19). Epstein-Barr virus (EBV) BHRF1 protein can counteract innate immunity by inducing mitochondria fission and facilitating their sequestration in mitophagosomes for degradation (20). Our previous study has confirmed that RSV-induced autophagy to promote viral replication and lung pathology (21). Although our results of transmission electron microscopy and confocal microscopy supported that RSV might induce mitophagy, more research should be done to confirm this. Considering the possibility of mitochondrial localization of RSV-NS1, in this study, we focused on RSV-NS1 protein to explore its effects on mitochondrial homeostasis and mitophagy.

Furthermore, we estimated the role of RSV-NS1-induced mitophagy in its antagonism of IFNβ induction. Besides, high throughout CoIP-MS technology was used to identify RSV-NS1 interacting protein, especially mitochondrial protein, to find out the possible molecular target of RSV-NS1 initiating mitophagy and hampering IFN responses. This study reveals how RSV-NS1 affects mitochondria dynamics, triggers mitophagy, and provides novel mechanisms via which RSV-NS1 antagonize IFN responses and new antiviral option by targeting RSV-NS1 interacting mitochondria protein.

## RESULTS

### RSV infection and RSV-NS1 protein could induce complete mitophagy in HEp-2 cells

**Fig 1:**
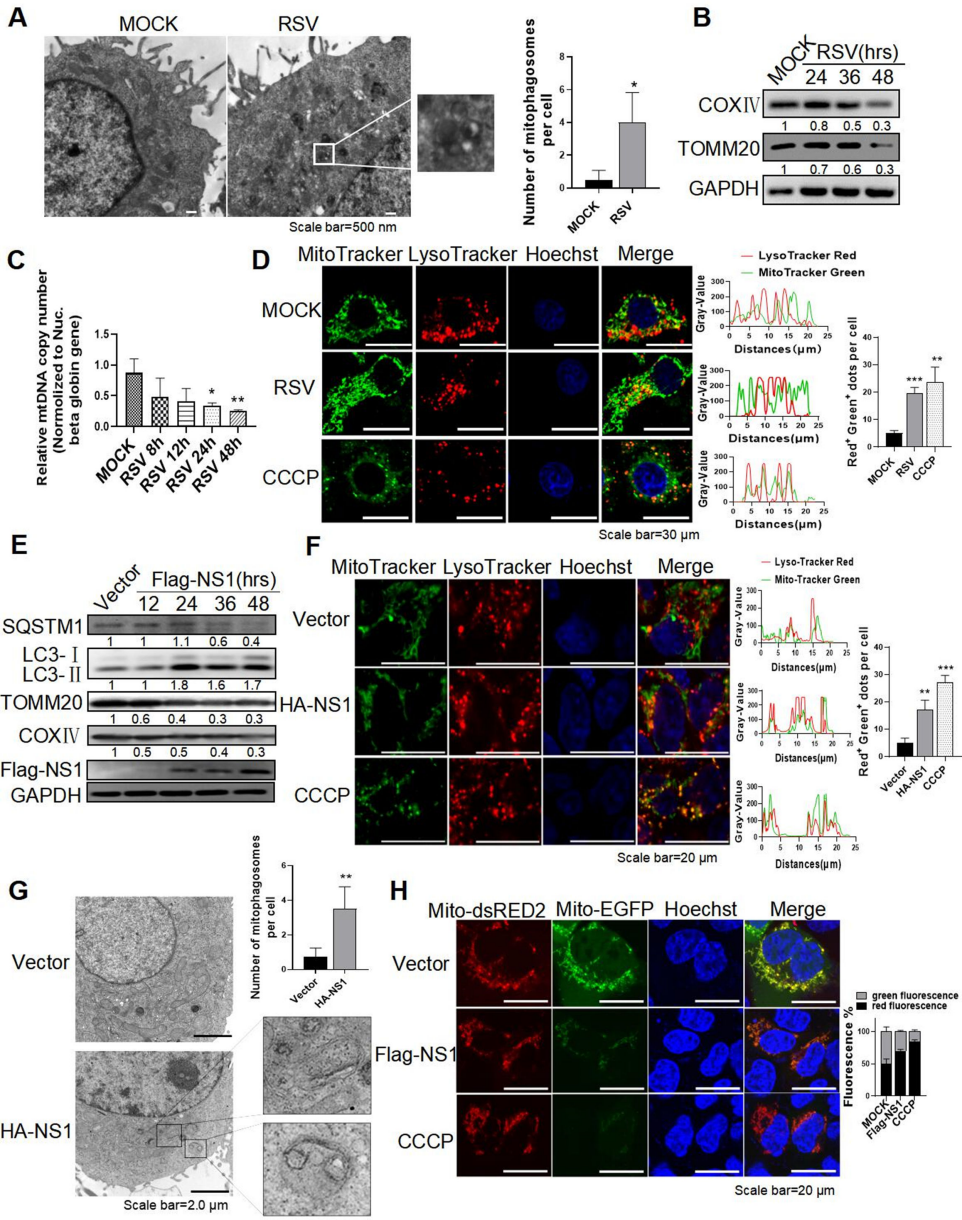
RSV infection and RSV-NS1 protein could induce complete mitophagy in HEp-2 cells. (**A–G**) HEp-2 cells were infected with RSV (MOI = 2) (**A–D**) or transfected with HA/Flag-NS1 plasmid (**E–G**) for indicated times. Mitophagosome (**A and G**), the relative expression of proteins (**B and E**), and the mitochondrial DNA (mtDNA) content normalized to single-copy nuclear gene (nDNA) HBB (mtDNA/nDNA ratios) (**C**), colocalization of mitochondria and lysosome stained with MitoTracker Green and LysoTracker Red, respectively (**D and F**) were detected by TEM, WB, qRT-PCR, and laser confocal microscope, respectively. The nuclei were stained with Hoechst. CCCP (20 µM) treatment for 6 h was used as a positive control. Scale bar = 500 nm (**A**)/30 µm (**D**)/20 µm (**F**)/2 µm (**G**). (**H**) After transfection with a mito-dsRED2-EGFP plasmid for 24 h, HEp-2 cells were transfected with Flag-vector or Flag-NS1 for another 30 h. Fluorescence was observed by laser confocal microscope. Each data represents the mean ± SD of three independent experiments. *: *P*＜0.05; **: *P*＜0.01; ***: *P*＜0.001.

Our previous study demonstrated that RSV could induce complete autophagy (21). However, it is still unclear whether RSV induces mitophagy. In this study, mitophagosomes, characterized by double-layered autophagic vacuoles containing swollen mitochondria, were observed in RSV-infected HEp-2 cells by transmission electron microscopy (TEM) (Fig. 1A). RSV infection reduced the expression of mitochondrial protein TOMM20 and COXIV, suggesting that RSV infection can reduce mitochondria mass resulting from mitophagy (Fig. 1B). In agreement with this observation, the qRT-PCR analysis showed that relative mitochondrial DNA (mtDNA) content (mtDNA/nDNA ratios) was decreased (Fig. 1C). Those results indicate that RSV could induce mitophagy. To further evaluate the effect of RSV infection on mitophagy flux, we analyzed the localization of mitochondria and lysosome labeled with MitoTracker Green and LysoTracker Red, respectively, and found an increase in colocalization of mitochondria and lysosome in RSV-infected cells, similar to that of positive control cells treated with carbonyl cyanide 3-chlorophenylhydrazone (CCCP) (Fig. 1D). CCCP, a mitochondrial oxidative phosphorylation uncoupler, was widely used to depolarize mitochondria and trigger DRP1-mediated mitochondria fragmentation and mitophagy (22). Besides, the cells infected with RSV displayed more red fluorescence of dsRED2 of Mito-dsRED2-EGFP reporter, similar to that of CCCP, indicating the delivery and degradation of mitochondria to lysosome (Fig. S1A). Those results confirmed the occurrence of complete mitophagy induced by RSV infection.

Next, we assessed whether the RSV-NS1 protein is involved in RSV-induced mitophagy. The result showed that RSV-NS1 significantly increased LC3-II expression and reduced TOMM20 and COXIV expression (Fig. 1E). Lipidated form of microtubule-associated protein 1 light chain 3 beta (LC3B), namely LC3-II, is a hallmark of autophagosome. Moreover, swollen mitochondria were trapped inside the autophagosome in HEp-2 cells transfected with pCAGGS-HA-NS1 plasmid by transmission electron microscopy (Fig. 1G). Besides, RSV-NS1-expressing cells displayed an increased number of GFP-LC3B dots and a decreased mtDNA content (Fig. S1B and C). These data indicated that RSV-NS1 protein could induce mitophagy. We next evaluated the effect of RSV-NS1 on autophagy flux, which was indicative of autophagic degradation activity. The expression of SQSTM1, an indicator of autophagic flux (23), was significantly decreased at 36and 48 h after transfection with pCAGGS-Flag-NS1 (Fig. 1E), indicating that autophagy flux is enhanced by RSV-NS1. Additionally, bafilomycin A1 (Baf A1), an autophagy flux inhibitor, significantly increased the expression level of LC3-II and SQSTM1 in HEp-2 cells transfected with a pCAGGS-Flag-NS1 plasmid (Fig. S1D). Similar to CCCP positive control, transfection with pCAGGS-Flag-NS1 increases the colocalization of mitochondria and lysosome (Fig. 1F), counterstained by MitoTracker Green and LysoTracker Red dye, respectively. Besides, exogenous NS1 protein caused a decrease in the green fluorescence of EGFP but not the red fluorescence of dsRED2 of Mito-dsRED2-EGFP reporter (Fig. 1H), indicating that RSV-NS1 protein can make the fusion of mitochondria with lysosomes. These results supported that RSV-NS1 induced complete mitophagy flux.

### RSV and RSV-NS1 protein could induce mitophagy by facilitating Drp1-dependent mitochondria fission

**Fig 2:**
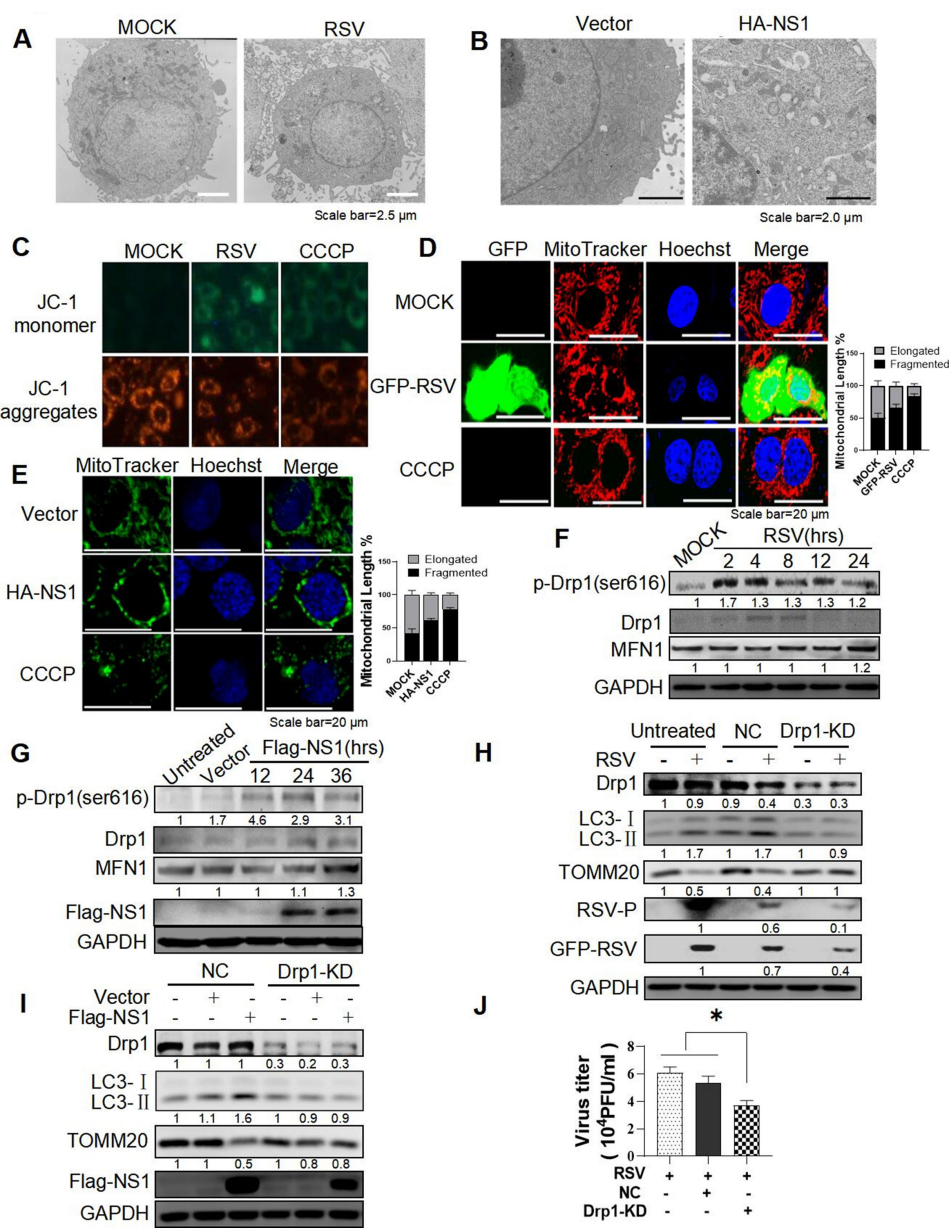
RSV and RSV-NS1 protein could induce mitophagy by facilitating Drp1-dependent mitochondria fission. (**A–C**) HEp-2 cells were infected with RSV (MOI = 2) (**A and C**) or transfected with a HA-NS1 plasmid for 36 h (**B**). The mitochondrial morphology (**A and B**) and mitochondrial membrane potential detected by JC-1 staining (**C**) were observed by TEM and fluorescence microscope, respectively. Scale bar = 2.5 µm (**A**)/2.0 µm (**B**). (**D–G**) HEp-2 cells were infected with GFP-RSV (MOI = 2) (**D and F**) or transfected with HA-NS1 plasmids for 36 h (**E and G**), and then stained with MitoTracker Red CMXRos (**D**) or MitoTracker Green (**E**), respectively. Nuclei were stained with Hoechst. The mitochondrial morphology and relative expression of proteins were observed by laser confocal microscopy and WB. Scale bar = 20 µm. (**H–J**) HEp-2, HEp-2-NC, and HEp-2-DRP1-KD cells were infected with RSV (**H and J**) or transfected with a Flag-NS1 plasmid for 36 h (**I**). Relative expression of proteins and viral titers were detected by WB and viral plaque assay, respectively. Each data represents the mean ± SD of three independent experiments. *: *P*＜0.05.

Except for the formation of mitophagosome, the number and morphology of mitochondria in HEp-2 cells infected with RSV or transfected with pCAGGS-NS1 plasmid were observed by TEM. RSV infection or RSV-NS1 protein decreased the number of mitochondria, accompanied by mitochondrial swelling, roundness, and disruption of mitochondrial crests (Fig. 2A and B). JC-1 probes were used to detect mitochondrial membrane potential (ΔΨm). Similar to CCCP positive control, RSV infection could attenuate the red signal while increasing the green signal (Fig. 2C). These results suggested that RSV infection or NS1 protein expression may alter mitochondrial dynamics, and finally disturb mitochondria function. It has been proposed that asymmetric fragmentation of mitochondria is closely linked to mitophagy (24). According to the results obtained by transmission electron microscopy, we speculated that RSV/RSV-NS1 protein might induce mitophagy by facilitating anomalous mitochondrial dynamics.

Next, we further confirmed the effect of RSV/RSV-NS1 on the mitochondria dynamics and assessed the role of mitochondria fission in mitophagy. First, the mitochondrial network morphology was dramatically altered in RSV-infected or RSV-NS1-expressing cells, changing from a tubular mitochondrial network to a highly fragmented network (Fig. 2D and E), similar to that of CCCP. In contrast to mitochondria homogenously distributed in the cytoplasm of control cells, abnormal and perinuclear aggregates of mitochondria were observed in RSV-infected or NS1-expressing cells (Fig. 2D and E). As mentioned above, mitochondria form a dynamic tubular network and continually undergo fusion and fission events. In mammals, the fusion of mitochondria’s outer membrane is mainly regulated by mitofusion (MFN) proteins, while mitochondria fission is majorly controlled by Dynamin-related protein 1 (DRP1) that acts as a marker for mitochondria dynamic (25–27). Evidence indicates phosphorylation plays a crucial regulatory role in DRP1 activity (28). Its phosphorylation at serine 616 is critical for its translocation from the cytosol to the mitochondria, which is an essential step at the beginning of mitochondrial fragmentation (26). Then we detected the effect of RSV infection or RSV-NS1 protein on the expression of MFN1 and DRP1. The results showed that the level of phosphorylated DRP1 at Ser616 was increased in RSV-infected or RSV-NS1-transfected cells, while the MFN1 level remained unchanged (Fig. 2F and G). These findings indicated that RSV infection or NS1 protein might cause DRP1-dependent mitochondria fission. To further assess the role of mitochondria fission in mitophagy, we used sgRNA lentivirus targeting DRP1. Knockdown of DRP1 (Fig. S2A) successfully abolished the higher expression of LC3-II and the lower expression of TOMM20 induced by RSV infection or pCAGGS-NS1 transfection (Fig. 2H and I). Treatment with Mdivi-1 at 10 µM/L, a selective inhibitor of DRP1 activity, exhibited similar effect (Fig. S2D and E) with no cytotoxicity (Fig. S2C). Those results demonstrate that RSV and NS1 proteins could induce mitophagy by facilitating DRP1-dependent mitochondria fission.

Since the importance of DRP1 in RSV-induced mitophagy, which is closely related to viral replication, we speculated that DRP1 might greatly affect RSV replication. To further clarify this, we tested the effects of Mdivi-1 or silencing DRP1 on GFP-tagged RSV replication. Knockdown of DRP1 or Mdivi-1 treatment reduced RSV-P, GFP, and M2-1 protein expression (Fig. 2H; Fig. S2D), total viral titers (Fig. 2J; Fig. S2F), and the number of green fluorescent cells (Fig. S2B andG). These results indicated that DRP1 played an important role in RSV replication.

### RSV-NS1-induced mitophagy was essential for its attenuated IFNβ response stimulated by RIG1 and RSV infection

**Fig 3:**
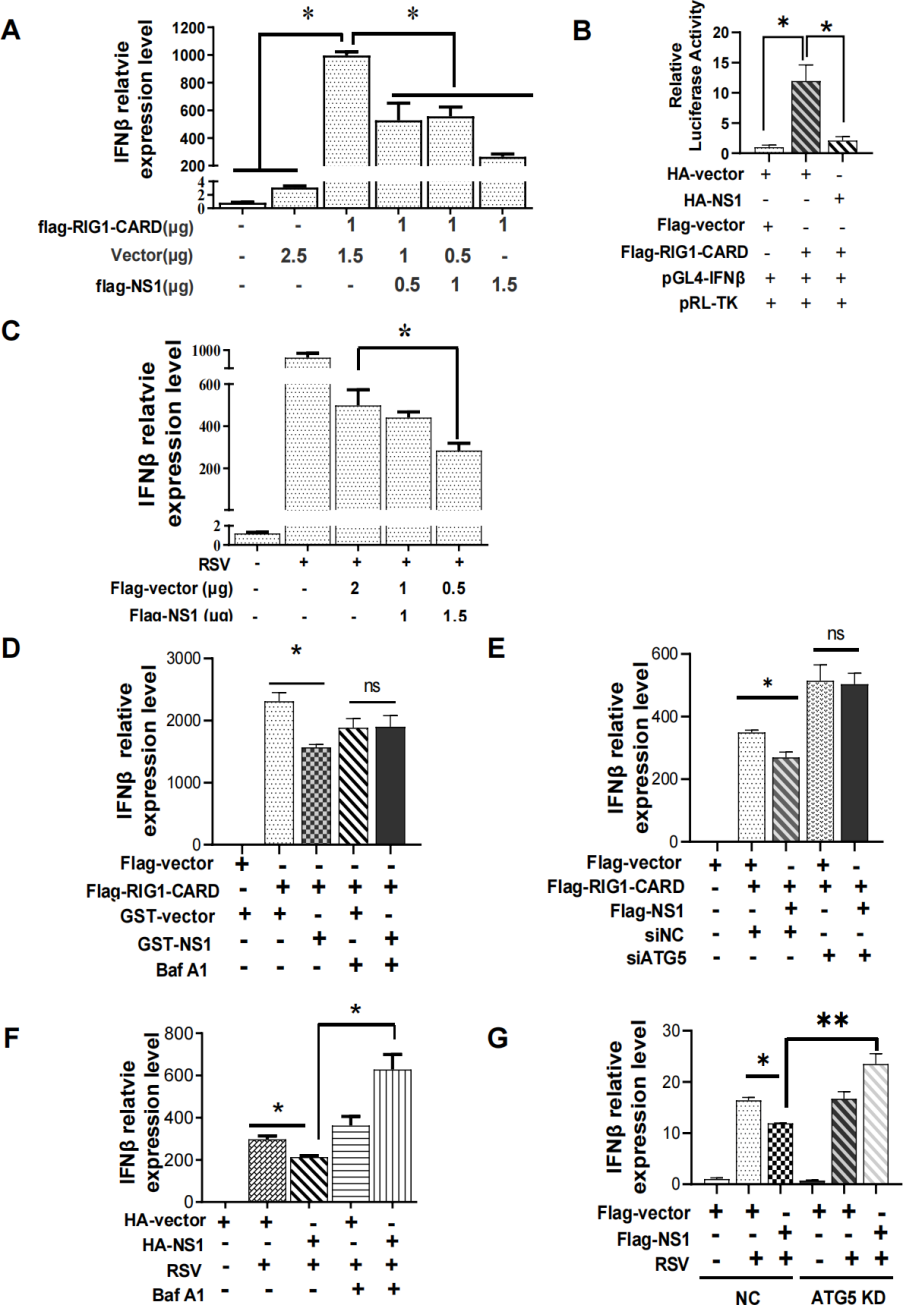
RSV-NS1-induced mitophagy was essential for its attenuated IFNβ response stimulated by RIG1 and RSV infection. (**A–D and F**) HEK 293 cells were co-transfected with indicated plasmids in the absence (**A–C**) or presence (**D and F**) of Baf A1 with (**C and F**) or without (**A, B, and D**) RSV infection. The IFNβ mRNA expression or IFN-β promoter activity was detected by qRT-PCR or Dual-Luciferase Reporter Assay, respectively. (**E and G**) Transient ATG5 knockdown HEK 293 cells (**E**) or HEp-2-ATG5-KD cells (**G**) were co-transfected with indicated plasmids with (**G**) or without (**E**) RSV infection. The IFNβ mRNA expression level was detected by qRT-PCR. Each data represents the mean ± SD of three independent experiments. *: *P*＜0.05; **: *P*＜0.01; ***: *P*＜0.001.

It has been proved that RIG1 is essential for interferon induction during RSV infection (29–31). In our study, to investigate the possible role of RSV-induced mitophagy in its suppression of IFN induction, we first confirmed the inhibitory effect of ectopically expressed RSV-NS1 on the RIG1- or RSV-driven IFNβ production. Transfection of RIG1 plasmid can significantly induce mRNA expression of IFNβ in HEK 293 cells, which can be decreased remarkably in cells expressing RSV-NS1 in a dose-dependent manner (Fig. 3A). The luciferase reporter assay showed that ectopically expressed RSV-NS1 protein could significantly inhibit luciferase activities of IFNβ promoter driven by RIG1 (Fig. 3B). To test the inhibition effect of RSV-NS1 on IFNβ mRNA induction in a more physiological context, we followed IFNβ mRNA induction upon RSV infection. Indeed, the level of IFNβ mRNA was also reduced in cells expressing the RSV-NS1 protein compared to control cells during RSV infection (Fig. 3C). All these results were consistent with that reported previously.

However, it is still obscure whether NS1-mediated mitophagy is involved in its inhibitory effect on IFNβ responses. To answer this question, we used autophagy inhibitor Baf A1 or siRNA or sgRNA lentivirus specifically targeting the autophagy-related gene ATG5 to block the autophagy process and assess whether they can abolish the inhibitory effect of RSV-NS1 on the IFNβ expression driven by RIG1 or RSV infection. The silencing efficiency of siATG5 or ATG5 sgRNA was validated by immunoblotting (Fig. S3A and B). The results showed that the inhibitory effect of RSV-NS1 on the RIG1- or RSV-induced IFNβ mRNA expression or protein level disappeared in cells pretreated with Baf A1 (Fig. 3D and F; Fig.S3C and D) or ATG5 deficient cells (Fig. 3E and G; Fig. S3E). In brief, these results indicated that RSV-NS1-induced mitophagy was crucial for its attenuated IFNβ response.

### RSV-NS1 could interact with mitochondria protein TUFM

**Fig 4:**
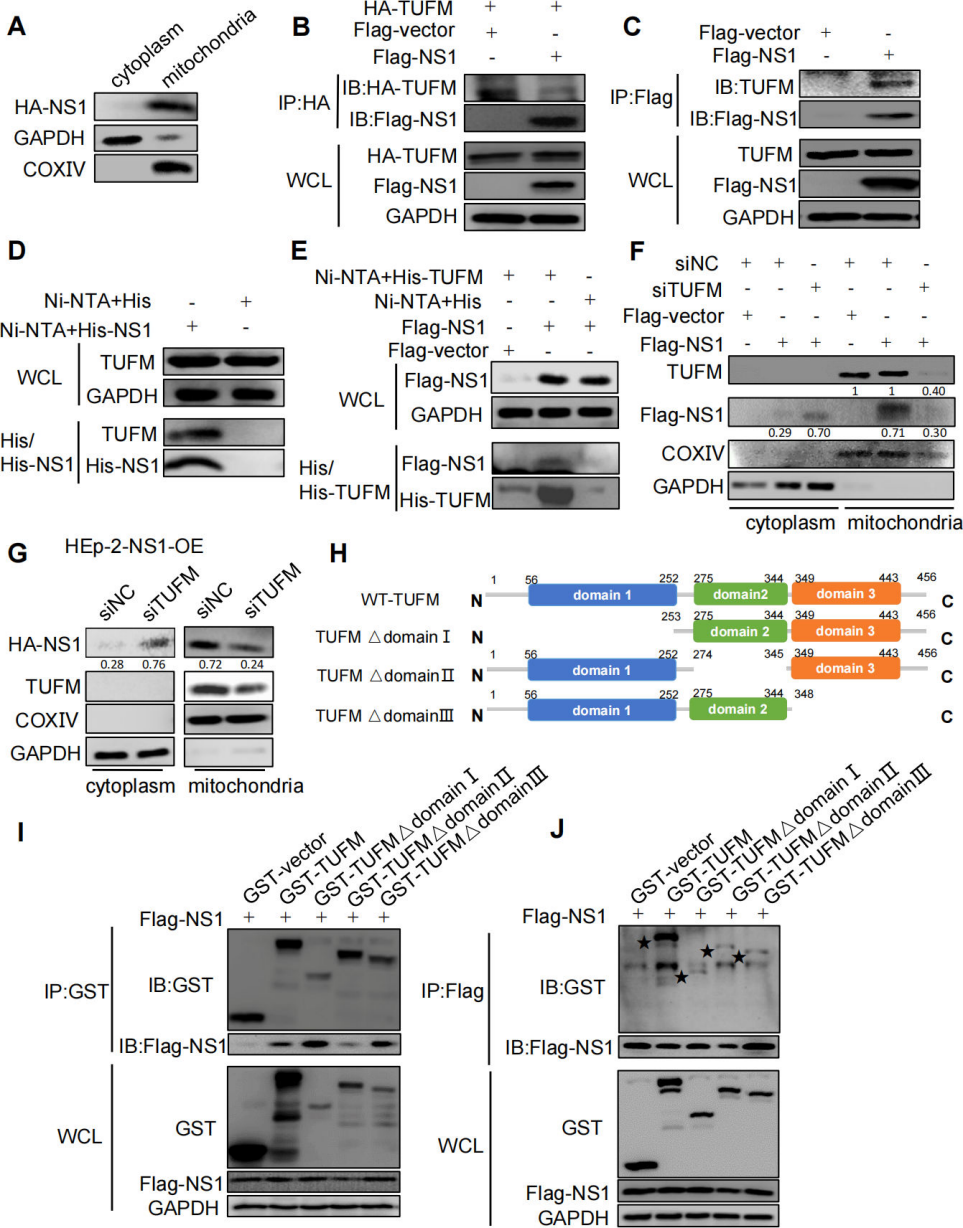
RSV NS1 could interact with mitochondria protein TUFM. (**A**) Cytosolic or mitochondrial localization of NS1 in stable NS1-expressing HEp-2 cells (HEp-2-NS1-OE) was analyzed via WB. (**B, C, I, and J**) HEK 293 cells were co-transfected with indicated plasmids for 48 h. Lysates were subjected to IP and analyzed via WB. (**D**) His or His-NS1 protein was incubated with lysates of HEK 293 cells. The mixture was subjected to Ni-NTA pulldown and analyzed via WB. (**E**) His or His-TUFM protein was incubated with lysates of HEK 293 cells, which was transfected with indicated plasmids for 48 h. The mixtures were subjected to Ni-NTA pulldown and analyzed via WB. (**F and G**) Transient NS1-expressing HEp-2 cells (**F**) or HEp-2-NS1-OE cells (**G**) were transfected with siNC or siTUFM for 16 h, mitochondrial and cytoplasmic proteins were analyzed via WB. Domain structures of wild-type TUFM and truncation mutants.

As mentioned above, RSV-NS1 protein can induce mitochondria fission and mitophagy. Previous studies hinted at the possibility of mitochondrial localization of RSV-NS1. It is reasonable to speculate that RSV-NS1 may affect mitochondria dynamics and mitophagy through its mitochondrial localization. First, we separated mitochondrial and cytoplasmic proteins from RSV-NS1 stable overexpression HEp-2 cells to observe its cellular distribution and found that RSV-NS1 protein was mainly accumulated on mitochondria and a small amount in the cytoplasm (Fig. 4A). Then we wondered whether RSV-NS1 could localize in mitochondria through interacting with mitochondrial proteins, which is critical for its ability to induce mitophagy and inhibit IFNβ production. To confirm that, cellular proteins associated with RSV-NS1 were collected by immunoprecipitation with flag antibodies from HEp-2 cells transfected with a pCAGGS-Flag-NS1 plasmid and identified by LC-MS/MS. A mitochondrial protein, Tu elongation factor, mitochondrial (TUFM), was identified in the candidate proteins. To confirm this interaction, we performed a series of reciprocal co-IP assays. It was found that HA-tagged TUFM could coprecipitate with flag-tagged NS1 (Fig. 4B). Besides, flag-NS1 could coprecipitate with endogenous TUFM protein (Fig. 4C). In line with this, His pulldown assay also showed that His-fused NS1 protein could pulldown endogenous TUFM (Fig. 4D). And His-fused TUFM protein could pulldown ectopic flag-tagged NS1 protein (Fig. 4E). Taken together, these data strongly confirmed the binding between NS1 and TUFM. To explore whether RSV-NS1 localized in mitochondria dependent on TUFM, we observed the effect of knockdown of TUFM with siRNA (Fig. S4A and B) on mitochondrial localization of NS1. As shown in HEp-2 cells that transiently (Fig. 4F) or stably express NS1 protein (Fig. 4G), the knockdown of TUFM resulted in an obvious decrease in the amount of NS1 protein in mitochondria and an increase of that in the cytoplasm. To further confirm these results, we generated TUFM knockout HEp-2 cells (HEp-2-TUFM-KO) with CRISPR/Cas9 (Fig. S4C). Colocalization of flag-NS1 with TOMM20 was also remarkably decreased in HEp-2-TUFM-KO cells (Fig. S4D). These data suggest that RSV-NS1 protein localized in mitochondria dependent on TUFM.

To map the essential domain in TUFM that is necessary for its interaction with RSV-NS1, we constructed TUFM domain I/II/III deleted mutants, namely pcDNA3-GST-TUFM Δdomain I, pcDNA3-GST-TUFM Δdomain II, and pcDNA3-GST-TUFM Δdomain III. The domain of TUFM is shown in Fig. 4H. The results of reciprocal co-IP showed that TUFM Δdomain I and TUFM Δdomain III could be able to precipitate RSV-NS1 comparable to WT TUFM, while TUFM Δdomain II resulted in a decrease in binding with NS1 (Fig. 4I). Although flag-NS1 could coprecipitate with WT, Δdomain I, Δdomain II, and Δdomain III TUFM, the amount of TUFM truncations precipitated decreased compared to WT-TUFM (Fig. 4J). These data indicated that the three domains of TUFM may be involved in the interaction with NS1.

### RSV or RSV-NS1 mediates TUFM-dependent mitochondria fission and mitophagy

**Fig 5:**
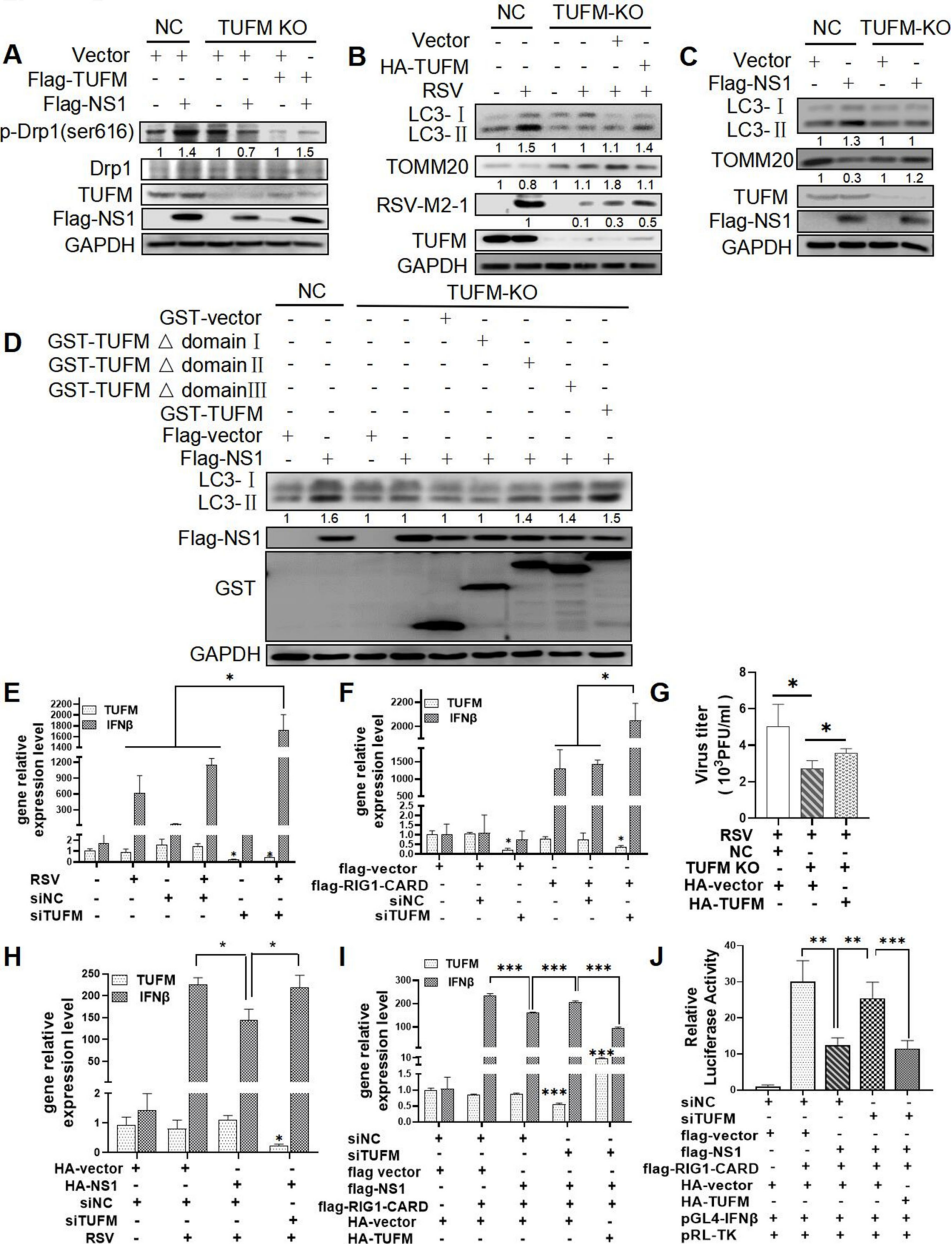
RSV or RSV NS1 could induce mitophagy and suppress IFN response by TUFM. (**A–D and G**) HEp-2-NC or HEp-2-TUFM-KO cells were co-transfected with indicated plasmids with (**B and G**) or without (**A, C, and D**) RSV infection. Relative expression of proteins and viral titers were detected by WB and viral plaque assay. (**E, F, I, and J**) HEK 293 cells were transfected with siNC or siTUFM for 16 h, then were mock-infected/infected with RSV for 24 h (**E**) or transfected with indicated plasmids for 36 h (**F, I, and J**). The IFNβ and TUFM mRNA expression and IFNβ promoter activity were detected by qRT-PCR and Dual-Luciferase Reporter Assay, respectively. (**H**) HEK 293 cells were co-transfected with indicated plasmids and siTUFM for 24 h, and then were mock-infected or infected with RSV for 24 h. The IFNβ and TUFM mRNA level were detected by qRT-PCR. Each data represents the mean ± SD of three independent experiments. *: *P*＜0.05; **: *P*＜0.01; ***: *P*＜0.001.

To explore whether TUFM was involved in regulating RSV or NS1-induced mitochondria fission and mitophagy, we knocked down TUFM expression and performed rescue experiments. RSV-NS1 lost its ability to increase the level of DRP1 phosphorylation in TUFM knockout cells, while ectopic expression of TUFM rescued this (Fig. 5A), implying that NS1 induced mitochondria fission dependent on TUFM. Moreover, knockdown of *TUFM* with siTUFM resulted in a decrease in LC3-II expression in RSV-infected or NS1-transfected HEp-2 cells (Fig. S5A and B). Similarly, knockout of TUFM resulted in the incompetence of RSV or NS1 to increase LC3-II expression and decrease TOMM20 expression (Fig. 5B and C). Rescue expression of WT-TUFM in HEp-2-TUFM-KO cells could partially reverse this ability of RSV (Fig. 5B) or NS1 (Fig. 5D lanes 5 and 9 and Fig. S5C), suggesting that RSV or RSV-NS1 protein induced mitophagy dependent on TUFM. Furthermore, similar to WT-TUFM, TUFM Δdomain II, and TUFM Δdomain III mutants, but not TUFM Δdomain I, could partially rescue the level of LC3-II protein expression blocked by *TUFM* knockout (Fig. 5D), hinting that N-terminal domain I of TUFM is critical for NS1-induced autophagy.

### RSV-NS1 mediates TUFM-dependent suppression of IFNβ response driven by RSV or RIG1

Previous studies reported that TUFM and RSV-NS1 could attenuate RLR-mediated innate antiviral signaling (10, 32). Next, we determined whether RSV-NS1-attenuated IFNβ response depends on TUFM. To clarify this, we first confirmed the role of TUFM in IFNβ regulation. Knockdown of *TUFM* with siRNA significantly increased IFNβ mRNA level induced by RIG1 or RSV (Fig. 5E and F), suggesting that TUFM can negatively regulate IFNβ. Second, inhibitory effects of RSV-NS1 on IFNβ expression induced by RSV or RIG1 disappeared in TUFM deficient cells (Fig. 5H and I; Fig. S5F), indicating that TUFM is essential for NS1-suppressed IFNβ response. Besides, IFNβ promoter luciferase reporter assay also showed that knockdown of TUFM reversed the inhibitory effects of RSV-NS1 on luciferase activities driven by RIG1 (Fig. 5J), supporting the essential role of TUFM in NS1-attenuated IFNβ response. The rescue experiment was performed in TUFM-knockdown cells and the results showed that ectopically expressing TUFM could rescue the inhibitory effect of NS1 on the RIG1-induced IFNβ mRNA expression (Fig. 5I), protein level (Fig. S5F), and IFNβ promoter activity (Fig. 5J), demonstrating that RSV-NS1 inhibit IFNβ response in TUFM-dependent mechanism. In brief, these results indicated that RSV-NS1 could induce autophagy and suppress IFNβ response dependent on TUFM.

Finally, we assessed the role of TUFM in RSV replication. Knockout of *TUFM* in HEp-2 cells resulted in a reduction in total viral titers (Fig. 5G), RSV M2-1 protein expression (Fig. 5B), RSV N and NS1 gene expression (Fig. S5E), and GFP fluorescence (Fig. S5D). Restoration of TUFM in HEp-TUFM-KO cells can partially rescue the level of these four indicators (Fig. 5B and G; Fig. S5D and E), further validating the pro-viral role of TUFM in RSV replication.

### RSV-NS1 protein may act as a potential autophagic adapter to induce PINK1-PARKIN-independent mitophagy by interacting with LC3B via its LIR motif

**Fig 6:**
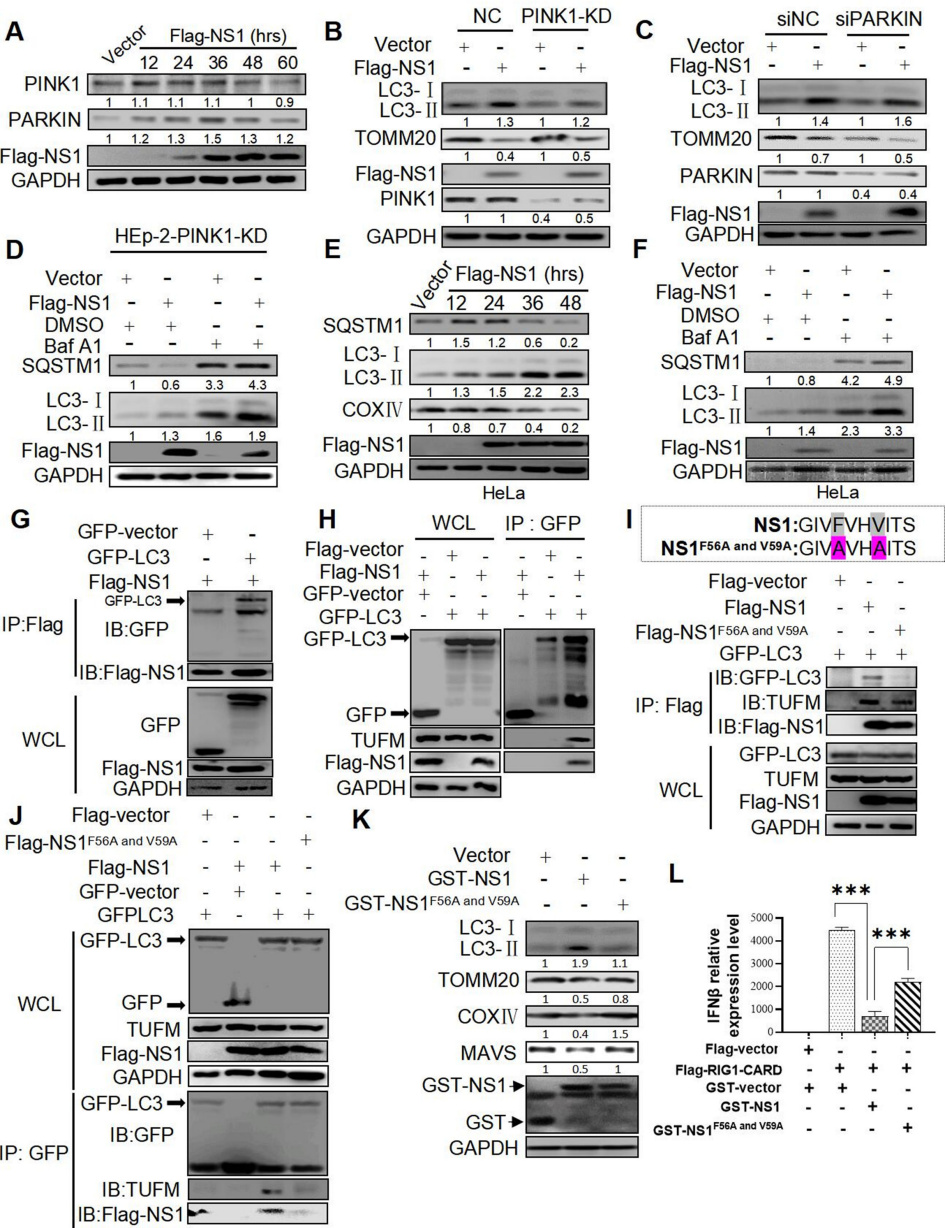
RSV NS1 may act as an autophagic recepter to induce PINK1-PARKIN-independent mitophagy by interacting with LC3B via its LIR motif. (**A and K**) HEp-2 cells were transfected with a Flag-NS1 plasmid for indicated times. Relative expression of indicated proteins was evaluated via WB. (**B–D**) HEp-2-PINK1-KD (**B and D**) or transient PARKIN knockdown cells (**C**) were transfected with indicated plasmids for 36 h in the absence or presence of BafA1. Lysates were evaluated via WB. (**E and F**) HeLa cells were transfected with a Flag-NS1 plasmid for 36 h in the absence (**E**) or presence (**F**) of Baf A1. Lysates were evaluated via WB. (**G–J and L**) HEK 293 cells were co-transfected with indicated plasmids for 48 h. Lysates were subjected to IP and analyzed via WB (**G–J**). The IFNβ mRNA expression was measured by qRT-PCR (**L**). Each data represents the mean ± SD of three independent experiments. ***: *P*＜0.001.

Although PINK1/PARKIN axis is the best-described mitophagy pathway, a growing body of evidence has shown that this pathway is not accountable for all mitophagy processes (33, 34). It is still unclear whether RSV-NS1 protein induces PINK1/PARKIN-dependent or -independent mitophagy. First, we detected the effect of ectopically expressing NS1 protein on the expression of PINK1 and PARKIN and found that it could enhance PARKIN but not PINK1 expression (Fig. 6A). Stable or transient knockdown of PINK1 or PARKIN in HEp-2 cells was performed with sgRNA lentivirus or siRNA (Fig. S6A and B), respectively, to define their roles in NS1-induced autophagy. PINK1 or PARKIN deficiency did not affect the ability of NS1 to increase LC3-II conversion and decrease TOMM20 expression (Fig. 6B and C), suggesting the dispensable role of PINK1/PARKIN in NS1-induced autophagy. Additionally, Baf A1 significantly increased the expression of LC3-II and SQSTM1 induced by NS1 in PINK1 deficient HEp-2 cells (Fig. 6D), further confirming the dispensable role of PINK1 in NS1-induced autophagy flux. Hela cells, which do not express endogenous PARKIN reported by others (35) and verified by our results (Fig. S6C), were used to study the role of PARKIN in mitophagy. As shown in Fig. 6E, RSV-NS1 expression could increase LC3-II conversion and decrease SQSTM1 and COXIV levels in HeLa cells. Baf A1 increased LC3-II and SQSTM1 expression levels in HeLa cells (Fig. 6F). Overexpression of PARKIN in HeLa cells could not change the expression level of LC3-II induced by NS1 (Fig. S6D). These data indicated that RSV-NS1 could induce mitophagy independently on PARKIN.

Mitophagy is a type of specific autophagy that sequesters mitochondria by autophagosomes through the interaction of autophagic adapters or receptors with LC3. Autophagic adapters can be recruited to the mitochondria in a ubiquitin-dependent or independent pathway and then target mitochondria to the autophagosomes via its LC3 interacting region (LIR) motif, which is critical for binding LC3 (36). Five major autophagic adapters, including OPTN, p62/SQSTM1, NBR1, NDP52/CALCOCO2, and TAX1BP1, have been identified (37, 38). Then we knock down the expression of these five proteins by CRISPR-Cas9 to assess their roles in RSV-NS1-induced mitophagy in HEp-2 cells. No matter which autophagic adapters we knocked down, NS1 could induce mitophagy, with an increase in LC3-II and a decrease in TOMM20 expression (Fig. S6E through N). The result indicated that these five major autophagic adapters might not be involved in NS1-triggered mitophagy. There may be one new LC3 adapter that may be involved in mitophagy induced by the NS1. Interestingly, we found a “canonical” LIR motif (W/F/YxxL/I/V) with which LC3B interacts in the NS1 protein sequence (56–59 aa, FVHV) (Fig. S6O). It has been confirmed that certain proteins with this motif may function as mitophagy receptors (39, 40), hinting the possibility of RSV-NS1 to act as an LC3 adapter to interact with LC3B. As predicted, flag-NS1 could coprecipitate with GFP-LC3B and vice versa (Fig. 6G and H). In line with this, His pulldown assay showed that recombinant *Escherichia coli* producing His-tagged NS1 protein coupling Ni agarose could pull down endogenous LC3B (Fig. S6P). The mutation of 56 and 59 residues in NS1 (namely as NS1^F56A and V59A^) blocks this interaction (Fig. 6I and J). Compared with wild NS1, NS1^F56A and V59A^ hardly precipitates GFP-LC3 (Fig. 6I), and vice versa (Fig. 6J). These results indicated that LIR motif of NS1 is essential for its binding with LC3B. As mentioned above, NS1 could interact with TUFM. We speculated that NS1 might combine with LC3 and TUFM to form a complex. In the precipitate of NS1 protein, LC3 and endogenous TUFM were detected (Fig. 6I lane 2), suggesting their potential interactions. Besides, LC3 could coprecipitate with TUFM in the presence, but not in the absence, of NS1 protein (Fig. 6I lanes 1 and 2 and Fig. 6J lanes 1 and 3). This result supported that NS1 may act as an adaptor protein to bridge the interaction of LC3 and TUFM. Furthermore, the amount of TUFM precipitated by GFP-LC3 is scarce in the presence of NS1^F56A and V59A^ (Fig. 6J lane 4), which may be due to a loss of adaptor function of NS1^F56A and V59A^ that could not bind with LC3. When the LIR motif of NS1 is mutated, it can interact with TUFM but not with LC3 (Fig. 6I lane 3 and Fig. 6J lane 4), suggesting that NS1 interacts with LC3 and TUFM through different motifs. Functionally, the mutation of 56 and 59 residues in NS1 reduced its ability to increase LC3-II expression and decrease TOMM20, COXIV, and MAVS expression (Fig. 6K). Besides, NS1^F56A and V59A^ could not inhibit IFNβ mRNA expression and protein level induced by RIG1 (Fig. 6L; Fig. S6Q). This implied the indispensable role of the LIR motif in NS1-mediated mitophagy and inhibition of IFN responses. NS1 may function as an adaptor protein to interact with mitochondrial protein TUFM and autophagosomal protein LC3B, target mitochondria to the autophagosomes, and finally induce mitophagy and inhibit IFN.

### TUFM can act as a regulator of RSV-induced autophagy and IFNβ response to favor viral replication and aggravate lung pathology *in vivo*

**Fig 7:**
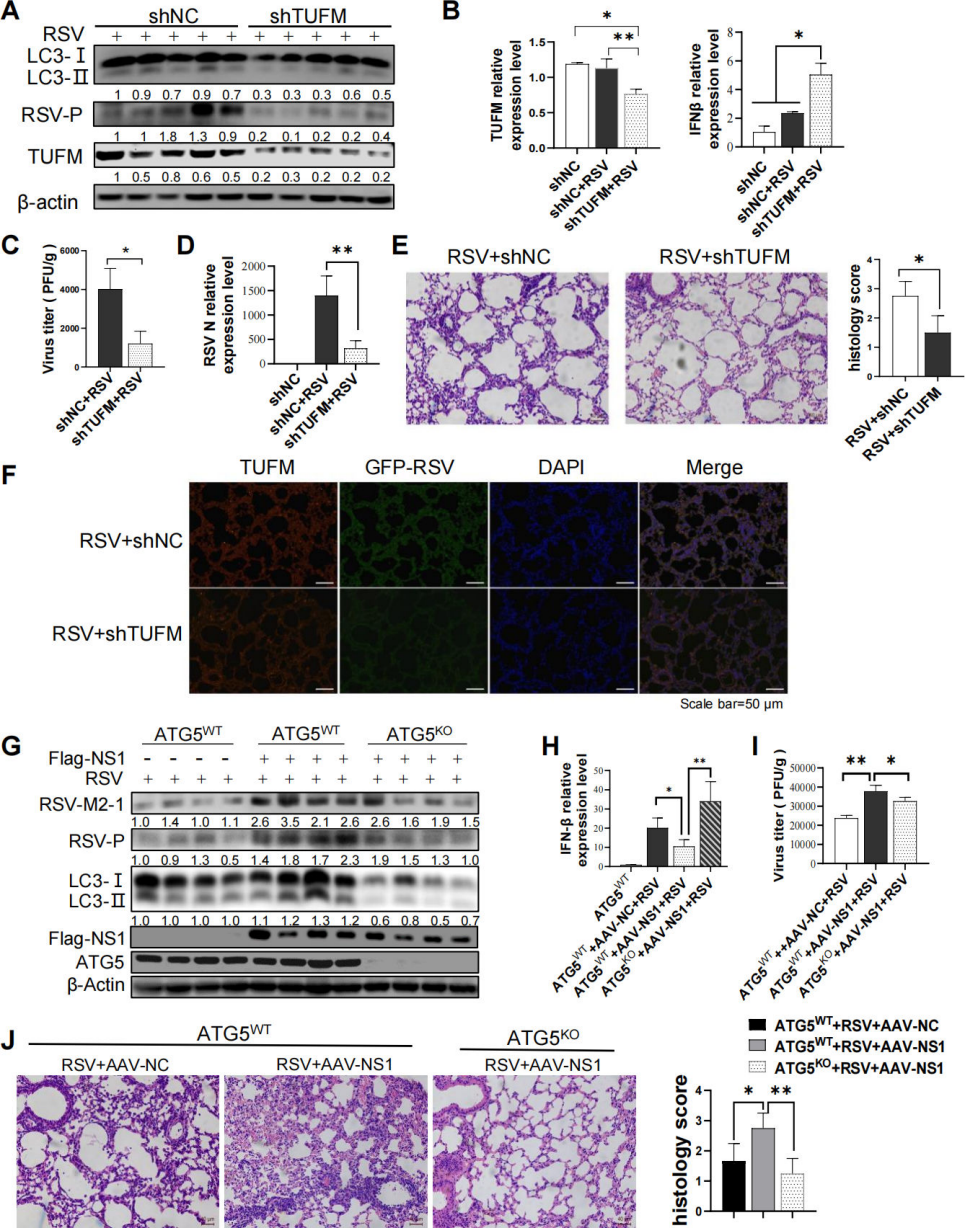
RSV NS1 inhibits IFNβ response by inducing TUFM-dependent autophagy *in vivo*. (**A–E**) Wide-type and TUFM knockdown Balb/c mice were generated and then infected with RSV (3  ×  10^6^ PFU per mouse) . Relative expression of indicated proteins (**A**) and IFNβ, TUFM (**B**), and RSV N (**D**) mRNA, viral titers (**C**), pathology (**E**), fluorescence intensity of GFP and TUFM protein (**F**) of mouse lungs were analyzed via WB, qRT-PCR, viral plaque assay, H&E staining, and IHF, respectively. Scale bar = 40 µm (**E**)/50 µm (**F**). (**G–J**) ATG5^WT^ or ATG5^KO^ C57BL/6 mice were infected with AAV-NC or AAV-flag-NS1 (1  ×  10^11^ AAV virions), followed by infection with RSV (3  ×  10^6^ PFU per mouse). Relative expression of indicated proteins (**G**) and IFNβ mRNA (**H**), viral titers (**I**), and pathology (**J**) of mouse lungs were analyzed via WB, qRT-PCR, viral plaque assay, and H&E staining, respectively. Scale bar = 40 µm (**J**). Results are means ± SD for five mice per group. *: *P*＜0.05; **: *P*＜0.01, ***: *P*＜0.001.

To confirm the role of TUFM in autophagy, IFNβ response, and RSV replication, we generated TUFM knockdown mice using adeno-associated viruses (AAV)-mediated shRNA transfer. The silencing efficiency of *TUFM* was validated by qRT-PCR (Fig. 7B), immune-blotting (Fig. 7A), and immunohistofluorescence (IHF) (Fig. 7F). Compared with that of WT mice at 3 days post-infection (dpi), LC3-II expression was lower (Fig. 7A), but IFNβ mRNA level was higher (Fig. 7B) in the lungs of the TUFM-KD mice. Similar results were observed at 5 dpi (data not shown). These data were consistent with the results *in vitro*, confirming the important roles of TUFM in autophagy and IFN regulation during RSV infection.

Next, we evaluated the role of TUFM in RSV proliferation and lung pathology. TUFM knockdown resulted in a decrease in viral P, NS2, NS1, M2, L, G, N, and M gene expression (Fig. 7D; Fig. S7), RSV P protein expression (Fig. 7A), total viral titers of mice lungs (Fig. 7C), and the lower GFP fluorescence intensity of GFP-RSV (Fig. 7F). In line with this, the hematoxylin and eosin (H&E) stain showed that lung pathology caused by RSV infection, which is characterized by lymphocyte infiltration and thickened alveolar septum, was ameliorated by TUFM knockdown (Fig. 7E). These results *in vivo* suggest that TUFM protein plays a positive and negative regulatory role in autophagy and IFNβ response, respectively, and favors RSV replication and lung pathology.

### RSV-NS1 could inhibit IFNβ response in an autophagy-dependent mechanism that promotes viral replication and aggravates lung pathology *in vivo*

To confirm the inhibitory role of RSV-NS1 in IFNβ response during RSV infection, we generated a recombinant adenovirus expressing the flag-NS1 fusion protein (AAV-flag-NS1). *ATG5^WT^* C57BL/6 mice were infected with AAV-flag-NS1 or AAV-NC by bronchial perfusion for two weeks, followed by RSV intranasal infection for 2 days. AAV-flag-NS1 mice with NS1 overexpression showed an increase in LC3-II protein expression (Fig. 7G) and a decrease in IFNβ mRNA expression (Fig. 7H) during RSV infection, which is consistent with the results *in vitro*. These results demonstrated that RSV-NS1 protein could inhibit RSV-induced IFNβ response. Besides, over-expressing flag-NS1 protein could significantly promote the expression of RSV M2-1 and P protein (Fig. 7G), increase viral titers (Fig. 7I), and aggravate the pathological injury of lung (Fig. 7J). AAV-NC mice lungs showed dilatation and hyperemia of pulmonary alveolar-capillary and infiltration of a small number of inflammatory cells, while there was a marked increase in the infiltration of inflammatory cells and thickness of alveolar septum in AAV-flag-NS1 mice lungs (Fig. 7J). Besides, IHF showed the colocalization of TUFM and flag-NS1 protein in the mice lungs (Fig. S8).

To further confirm whether RSV-NS1 inhibited IFNβ response in autophagy-dependent mechanism, mice with lung-specific deficiency of *ATG5* (*ATG5*^F/F, Sftpc-cre^, named as ATG5^KO^ C57BL/6) and their control littermates (*ATG5* ^F/F^, named as *ATG5*^WT^ C57BL/6) were generated. ATG5^KO^ or ATG5^WT^ C57BL/6 mice were infected with AAV-flag-NS1 for 2 weeks, followed by RSV intranasal infection. ATG5 deficiency abolished or decreased the inhibitory effect of RSV-NS1 on IFNβ expression (Fig. 7H) and exhibited a lower RSV replication (Fig. 7G and I), indicating an important role of autophagy in NS1-mediated IFNβ escape and pro-viral effect. In line with this, RSV-NS1 could not aggravate lung pathology in ATG5^KO^ C57BL/6 mice (Fig. 7J). In brief, these data supported that RSV-NS1 could inhibit IFNβ response, promote viral replication, and worsen lung pathology in the autophagy-dependent mechanism.

## DISCUSSION

Understanding RSV and mitochondrial interactions may provide valuable insights into viral pathogenesis and a novel therapeutic target for RSV infection. Our study comprehensively evaluated RSV infection on mitochondria dynamics and mitophagy and a possible link between them. Mdivi-1 or sgDRP1 lentivirus used to inhibit mitochondria fission can successfully abolish mitophagy triggered by RSV, demonstrating the critical role of mitochondria fission in RSV-induced mitophagy. However, a detailed study may require identifying the viral protein involved and its crucial mitochondrial protein targets.

RSV NS proteins, the earliest and most abundantly expressed viral protein, are known to suppress IFN pathways. Intriguingly, its activity requires the assembly of a large NS degradosome (NSD) complex on the mitochondria that comprise NS1 protein with several unknown host factors (9). Our study confirmed mitochondria localization of RSV-NS1 protein. Besides, we used various technologies to demonstrate that NS1 expression alone could mediate mitochondria fission and complete mitophagy. Like RSV infection, ectopically expressed NS1 protein increased the phosphorylated DRP1 at Ser616. However, it is worth noting that the significant increase of Drp1 phosphorylation was shown at 2 h post-infection and at 12 h post-transfection, where NS1 expression appears to be quite low, we cannot rule out the possibility that other factors, such as other viral structural proteins, virus adsorption process, or the stress stimuli of transfection, may increase the Drp1 phosphorylation. Next, Mdivi-1 or Knockdown of DRP1, almost completely blocked the higher expression of LC3-II and the lower expression of TOMM20 induced by NS1 protein. These data implied that the NS1 protein could induce mitophagy by facilitating DRP1-dependent mitochondria fission. Taking into account that fragmentation of mitochondria may dampen MAVS-mediated IFN signaling (41) and the pro-viral role of autophagy in RSV or many other viruses (21, 42, 43), DRP1 may act as a potential target for antiviral agents. In our study, we observed that knockdown of DRP1 led to a decline in RSV replication. Similar results were reported in CSFV infection (44). However, more *in vivo* studies are needed to determine whether it can be used as a potential antiviral agent. In addition, this finding does not exclude the possibility that other viral proteins may localize in the mitochondria and trigger mitochondria fission and mitophagy. SILAC-based quantitative proteomic analysis has identified that other viral proteins, such as M and P proteins, are present in mitochondrial fractions from RSV-infected cells (45). More studies are needed to verify this proteomic result and clarify their roles in RSV-induced mitochondria fission and mitophagy.

Mitophagy has been considered as a central mechanism for regulating innate antiviral responses. Many viruses, such as measles virus and EBV, have been reported to hijack mitophagy to restrain host innate immune for their pathogenesis (20, 46). Our study observed that RSV-NS1 protein could induce mitophagy and inhibit IFN responses *in vitro* or *in vivo*. However, whether mitophagy is associated with NS1-suppressed IFN responses is obscure. In our study, blocking autophagy with Baf A1, siRNA targeting *ATG5*, or sgRNA lentivirus targeting *ATG5* abolished the inhibitory effect of RSV-NS1 on the RIG1- or RSV-driven IFNβ expression. Data from ATG5 knockout mice also confirmed the *in vitro* findings. These results supported that NS1 could inhibit IFNβ dependent on autophagy.

The IP-LC-MS/MS was used to identify possible mitochondrial proteins that might interact with RSV-NS1 and participate in NS1-mediated mitophagy and immune evasion, and peptide profiles matched TUFM were identified. TUFM is a highly conserved GTPase localized in mitochondria. It has been implicated in various biological processes, including mitochondrial protein translation elongation and biosynthesis, oxidative phosphorylation, oncogenesis, and protein quality control (47–51). TUFM also plays an important role in regulating type I IFN response and autophagy. TUFM can recruit ATG5-ATG12 and NLRX1 and shows dual functions in inhibiting RIG1 signaling and promoting autophagy (32). We speculated that TUFM might play crucial roles in NS1-mediated mitophagy and suppression of IFNβ to promote virus replication. Our study provided strong evidence to confirm the interaction between NS1 and TUFM. Knocking down or out TUFM significantly decreased the localization of NS1 in mitochondria, decreased the levels of phosphorylated DRP1 and mitophagy, and reduced NS1’s inhibitory effect on IFNβ. Restoration of TUFM can partially rescue the above abilities of NS1. These results confirm our hypothesis that NS1 could induce mitochondria fission and mitophagy and suppress IFNβ response dependent on TUFM.

Similarly, some other viral proteins, such as the M protein of human parainfluenza virus type 3 (HPIV3), Gn protein of Hantavirus (HTNV), PB1-F2 protein of influenza A virus (IAV), translocate to mitochondria by interacting with TUFM, and then recruit LC3B to trigger mitophagy and finally attenuate IFN responses (39, 40, 52). TUFM will be a potential target for developing universal antiviral drugs. In our study, knockdown of TUFM exhibited anti-RSV roles *in vitro or in vivo*.

TUFM has three domains, including an N-terminal GTP-binding domain (domain I), domain II, and a C-terminal domain (domain III), among which, domain I contains a mitochondria targeting sequence (MTS) (53). Besides, domain I was previously shown to mediate the interaction with other proteins, such as ATG5-ATG12 conjugate and PB1-F2 protein of IAV (32, 39). Unlike their studies, our study indicates that three domains of TUFM may be involved in interacting with the RSV-NS1. The absence of each domain reduced the binding between them, which seems to be more dependent on the spatial conformation. X-ray crystallography was needed to verify this. Furthermore, functional studies showed the critical role of domain I in the NS1-induced autophagy. We speculated that although TUFM with the deletion of domain I can interact with NS1, the absence of the MTS sequence may lead to a decrease in the mitochondrial localization of NS1 and, finally, loss of the ability to trigger mitophagy, which hints an indispensable role of mitochondrial localization of NS1 in mitophagy induction.

TUFM protein has been identified as a substrate of PINK1, which can regulate mitophagy by phosphorylating TUFM (54, 55). So PINK1/PARKIN pathway may be involved in NS1-mediated mitophagy. However, our results excluded this possibility. Neither knocking down PINK1 or PARKIN in HEp-2 cells nor using HeLa cells with endogenous PARKIN deficient affected autophagy induced by NS1. Besides, the knockdown of known mitophagy receptors does not affect NS1-induced mitophagy, hinting at the dispensable role of these autophagic adapters in NS1-triggered mitophagy. However, SQSTM1 expression was significantly decreased in NS1-expressing cells, which was significantly increased in the presence of Baf A1, indicating that SQSTM1 may be involved in the NS1-induced autophagy, which seems to be contradictory to the phenomenon we just mentioned. NS1 may induce mitophagy through two separate mechanisms: SQSTM1-dependent and SQSTM1-independent mechanisms. Regardless of the mechanism, NS1 initiated PINK1/PARKIN independent mitophagy, in which some mitochondrial proteins, such as FUNDC1 and Bnip3, normally have a classical LIR motif (F/W/YxxL/I/V) to interact with LC3 and function as mitophagy receptors (36, 56, 57). Intriguingly, RSV-NS1 carried a typical LIR motif F(56)xxV(59). NS1 may act as a new LC3 adaptor to bind LC3B and TUFM and induce mitophagy. WT NS1 could physically interact with endogenous and exogenous LC3B, while NS1^F56A and V59A^ failed, hinting the essential role of the LIR motif of NS1 in its interaction with LC3B. Besides, in the precipitate of NS1 protein, LC3B and TUFM were both presents, implying that these three proteins form complexes. The absence of WT NS1 or the mutation of the NS1 LIR motif blocks the interaction between LC3B and TUFM, supporting our hypothesis that NS1 may act as an adaptor protein to bridge the interaction between LC3 and TUFM. Although NS1^F56A and V59A^ can still interact with TUFM, its function to induce mitophagy and inhibit IFNβ was greatly impaired, hinting that the interaction between NS1 and LC3, as well as TUFM, is indispensable in NS1-mediated mitophagy and inhibitory effect on IFN response. Our results found that a viral protein, RSV-NS1 protein, may function as a new mitophagy adaptor to bridge the autophagosome and mitochondria by interacting with LC3B and TUFM to induce mitophagy.

In summary, this study reveals a novel mitochondrial target with which RSV-NS1 interacts, a possible new role of NS1 protein, which may act as a new mitophagy adaptor, and the molecular mechanism via which NS1 affects mitophagy and antagonizes IFN responses. Our study provides a novel insight into the relationship between viral protein, host mitochondria, and IFN regulation and the discovery of a new antiviral option by targeting NS1-TUFM interactions.

## MATERIALS AND METHODS

### Animals and treatment

Four-week-old female specific-pathogen-free (SPF) BALB/c mice were purchased from the Experimental Animal Center of Hebei Medical University. All mice were housed in temperature-controlled individual ventilated cages (IVCs) with a 12-h light/12-h dark cycle and were fed standard chow and sterile tap water. All experimental procedures were performed in compliance with institutional animal welfare guidelines and were carried out according to the criteria outlined in the *Guide for the Care and Use of Laboratory Animals* and with the approval of the Animal Care and Use Committee of Hebei Medical University.

#### The generation of TUFM knockdown mice by using adeno-associated virus serotype 9 expressing short hairpin RNA

The mice were infected with AAV9-mCherry-shNC (20 µL containing about 1  ×  10^11^ AAV virions, produced by GENECHEM) or AAV9-mCherry-shTUFM by bronchial perfusion (20 µL containing about 1  ×  10^11^ AAV virions, produced by GENECHEM) for 2 weeks to produce control mice or TUFM knockdown mice, respectively.

#### Atg5-KO mice

ATG5^flox/flox^ mice were provided by the RIKEN BRC through the National Bio-Resource Project of the MEXT, Japan, and kindly provided by Quan Chen, Institute of Zoology, Chinese Academy of Sciences. Lung-specific *ATG5* conditional knockout mice (*ATG5^flox/flox;cre+^*, namely *ATG5*^KO^) were generated by mating Atg5^flox/flox^ mice with Sftpc-Cre mice (purchased from ViewSolid Biotech, Beijing, China). Lung-specific knockout of *ATG5* was induced by intraperitoneal injection of tamoxifen (Sigma-Aldrich) at a dose of 75 mg/kg of body weight, once every 24 h for a total of 7 consecutive days. There is a 7-day waiting period for Cre characterization work between the final injection and necropsy/histological analysis. Wild-type littermates (ATG5^flox/flox; cre−^, namely ATG5^wt^) were used as control mice.

#### The generation of RSV NS1 overexpressing mice using AAV6-mediated gene delivery

Wild-type littermates (*ATG5*^wt^) were infected with AAV6-NC (20 µL containing about 1  ×  10^11^ AAV virions, produced by GENECHEM) or AAV6-Flag-NS1 (20 µL containing about 1  ×  10^11^ AAV virions, produced by GENECHEM) for 2 weeks to produce control mice or RSV NS1 overexpressing mice, respectively. *ATG5*^KO^ mice were infected with AAV6-Flag-NS1 to overexpress NS1 protein in *ATG5*^KO^ mice.

#### Animal grouping

To detect the role of TUFM in RSV-induced autophagy, IFNβ response, and viral replication, control mice (*n*  =  10) or TUFM knockdown mice (*n*  =  10) were infected with RSV-GFP (3  ×  10^6^ PFU per mouse) intranasally. Each group was randomly divided into two subgroups (5 mice/subgroup), and the mice of each subgroup were sacrificed on 3 or 5 days after treatment. The silencing efficiency of TUFM was validated by quantitative real-time PCR (qRT-PCR), western blot, and IHF. LC3-II protein expression was detected by western blotting. IFNβ in mouse lungs was detected by qRT-PCR. Viral genes, RSV P protein, GFP fluorescence intensity of GFP-RSV, and viral titers in mouse lungs were detected by qRT-PCR, western blotting, HIF, and plaque assay, respectively, to ascertain RSV replication. Lung pathology was detected by H&E stain. Control mice treated with an equal amount of phosphate-buffered saline (PBS) intranasally were set as a negative control group.

To confirm the essential role of autophagy mechanism in the inhibitory effect of RSV NS1 in IFNβ response during RSV infection, *ATG5*^wt^ (*n*  = 4), *ATG5*^wt^ mice (*n*  =  4), and *ATG5*^KO^ mice (*n*  =  4) were infected with AAV6-NC (20 µL containing about 1  ×  10^11^ AAV virions, produced by GENECHEM), AAV6-Flag-NS1 (20 µL containing about 1  ×  10^11^ AAV virions, produced by GENECHEM), and AAV6-Flag-NS1, respectively. Then, we infected the three groups of mice with RSV-GFP (3  ×  10^6^ PFU per mouse) intranasally for 2 days. The overexpression of NS1 and the knockout of ATG5 were validated by western blot. LC3-II protein expression was detected by Western blotting. IFNβ in mouse lungs was detected by qRT-PCR. The expression of RSV P and M2-1 protein and viral titers were examined by Western blotting and plaque assay, respectively, to evaluate RSV replication. Lung pathology was detected by H&E stain. IHF was performed to detect the colocalization of NS1 and TUFM.

### Antibody, reagents, and plasmids

All the antibodies, plasmids, and reagents applied here are listed in the Table 1.

**TABLE 1:** List of antibodies, plasmids and other reagents applied in the study

	Source	Identifier
Antibodies		
Anti-COX IV antibody [EPR9442(ABC)]	Abcam	ab202554
Anti-TUFM antibody [EPR12797(B)]	Abcam	ab173300
Anti-respiratory syncytial virus M2-1 protein antibody [RSV5H5]	Abcam	ab94805
Anti-respiratory syncytial virus phosphoprotein antibody [RSVH102]	Abcam	ab94965
TUFM rabbit pAb	ABclonal Technology	A6423
ATG5 antibody	Abways Technology	CY5766
GST antibody	Abways Technology	AB0055
GFP-Tag mouse monoclonal antibody	Abways Technology	AB0005
DRP1 antibody	Affinity Biosciences	DF7037
SQSTM1/p62 (D5L7G) mouse mAb	Cell Signaling Technology	88588
LC3A/B (D3U4C) XP rabbit mAb	Cell Signaling Technology	12741
Phospho-DRP1 (Ser616) (D9A1) rabbit mAb	Cell Signaling Technology	4494
Anti-DDDDK-tag mAb	Medical & Biological Laboratories	M185-3L
Anti-HA-tag mAb	Medical & Biological Laboratories	M180-3
Anti-GST-tag mAb	Medical & Biological Laboratories	M209-3
Anti-His-tag mAb	Medical & Biological Laboratories	D291-3
PINK1 antibody	Novus	BC100-494
GAPDH polyclonal antibody	Proteintech Group	10494–1-AP
TOM20 polyclonal antibody	Proteintech Group	11802–1-AP
MFN1 polyclonal antibody	Proteintech Group	13798–1-AP
PARK2/Parkin monoclonal antibody	Proteintech Group	66674–1-Ig
Beta actin monoclonal antibody	Proteintech Group	66009–1-Ig
NDP52 monoclonal antibody	Proteintech Group	66401–1-Ig
NBR1 polyclonal antibody	Proteintech Group	16004–1-AP
Optineurin rabbit mAb	Zen BioScience Co., Ltd.	R27111
TAX1BP1 rabbit mAb	Zen BioScience Co., Ltd.	R25967
Plasmids		
pGL4-IFNβ-luc	Constructed in our lab	N/A
mito-dsRED2-EGFP	MiaoLingBio	P4954
pCAGGS-Flag	Institut Pasteur of Shanghai, Chinese Academy of Sciences	
pCAGGS-Flag-NS1	Constructed in our lab	N/A
pCAGGS-Flag-NS1^F56A and V59A^	Constructed in our lab	N/A
pCAGGS-Flag-RIG1	Constructed in our lab	N/A
pCAGGS-HA	Institut Pasteur of Shanghai, Chinese Academy of Sciences	
pCAGGS-HA-NS1	Genscript	
pCDNA3.1-GST	Lab stored	
pCDNA3.1-GST-NS1^F56A and V59A^	Constructed in our lab	N/A
pCDNA3.1-GST-TUFM	Constructed in our lab	N/A
pCDNA3.1-GST-TUFM△domain1	Constructed in our lab	N/A
pCDNA3.1-GST-TUFM△domain2	Constructed in our lab	N/A
pCDNA3.1-GST-TUFM△domain3	Constructed in our lab	N/A
pCMV-HA	Lab stored	
pCMV-HA-TUFM	Sino Biological	HG16801-NY
pET-28a-his-TUFM	Constructed in our lab	N/A
pEX3-GFP-LC3	GenePharma	
PX459-puro-sgTUFM	Constructed in our lab	N/A
pGL4-TK	Constructed in our lab	N/A
pL-CRISPR.GFP-PINK1-1	Constructed in our lab	N/A
pL-CRISPR.GFP-PINK1-2	Constructed in our lab	N/A
pL-CRISPR.GFP-PINK1-3	Constructed in our lab	N/A
pL-CRISPR.GFP-PINK1-4	Constructed in our lab	N/A
Ycas-LV001	Ubigene Biosciences	
hDNM1L gRNA1 KO plasmid	Ubigene Biosciences	YKO-LV013-hDNM1L[gRNA1]
hNBR1 gRNA2 KO plasmid	Ubigene Biosciences	YKO-RP003-hNBR1[gRNA2]
hCALCOCO2 gRNA2 KO plasmid	Ubigene Biosciences	YKO-RP003-hCALCOCO2[gRNA2]
hOPTN gRNA1 KO plasmid	Ubigene Biosciences	YKO-RP003-hOPTN[gRNA1]
hSQSTM1 gRNA3 KO plasmid	Ubigene Biosciences	YKO-RP003-hSQSTM1[gRNA3]
hTAX1BP1 gRNA1 KO plasmid	Ubigene Biosciences	YKO-RP003-hTAX1BP1[gRNA1]
Chemical reagents		
Bafilomycin A1	Cayman	11038
Mdivi-1	MedChemExpress	HY-15886
Lipofectamine 2000	Invitrogen	11668019
Carbonyl cyanide 3-chlorophenylhydrazo	Solarbio	C6700

### Cells and the virus

Vero E6 and HEK 293 cells were cultured in Dulbecco’s modified Eagle’s medium (12800-58; Gibco) supplemented with 10% fetal bovine serum (FBS, 34894428S; Biological Industries), 10 mM HEPES (0511; Biosharp, Amresco), and 0.1% penicillin-streptomycin solution (P1400; Solarbio) in a humidified 5% CO_2_ atmosphere at 37°C. HEp-2 and Hela cells were grown in RPMI 1640 medium (31800-022; Gibco) supplemented with 10% FBS, 10 mM HEPES, and 0.1% penicillin-streptomycin solution in a humidified 5% CO_2_ atmosphere at 37°C. Respiratory syncytial virus A strain Long from ATCC and GFP-RSV A strain, kindly provided by Professor He Jinsheng (Beijing Jiaotong University), were amplified in Vero E6 cells. Viruses were harvested from the culture supernatant of Vero E6 cells at 72–96 h post-infection and stored at −80°C until use.

### Viral infection and virus titer assays

HEp-2 cells were infected with RSV as indicated or mock-infected with PBS for 2 h with serum-free RPMI 1640 and shaking every 15 min. After washing with 1× PBS, the cells were cultured with RPMI 1640 medium containing penicillin (100 U/mL), streptomycin (100 µg/mL), and 2% FBS until they were harvested.

RSV titers were determined using a plaque assay. Briefly, 10-fold serial dilutions of RSV-infected cell lysates or lung homogenates were incubated with Vero E6 cells in 96-well plates for 2 h with shaking every 15 min. Then, supernatants were removed and replaced with 1% (wt/vol) methylcellulose semisolid culture medium at 37°C in a CO_2_ incubator for 4–7 days. Plaques or the number of GFP signals in Vero E6 monolayers were counted by microscope. The RSV titer is expressed in PFU/mL cell lysates or PFU/g lung.

### Western blot analysis

Cells were washed two times with ice-cold PBS and then lysed with radioimmunoprecipitation assay (RIPA) lysis buffer (P0013; Beyotime) supplemented with 1 mM phenylmethylsulfonyl fluoride (PMSF, BL507A; Biosharp) and phosphatase inhibitors (P1260; Solarbio) on ice for 30 min. The supernatants were collected by centrifugation at 12,000 rpm for 15 min at 4°C, and the protein concentration was determined by NanoDrop 2000c spectrophotometer (EW-83061-12; Thermo Scientific). Equal amounts of total protein, cytoplasmic, or mitochondrial protein were separated by SDS-PAGE and transferred onto polyvinylidene fluoride membranes (PVDF, Millipore). After being blocked with 5% nonfat milk in 1× TBS-Tween 20, the membrane was incubated with primary antibodies overnight at 4°C. After washing, the membrane was incubated with HRP-conjugated secondary antibody for 1 h at room temperature (RT). Finally, the membranes were washed, developed with a Western Lightning plus-ECL reagent (NEL104001EA; PerkinElmer), and detected with a Synoptics Syngene Bioimaging instrument (R114075; Synoptics). GAPDH or β-actin was used as a loading control for immunoblotting. The relative expression of proteins was quantified by densitometry using ImageJ software.

### Cell transfection, fluorescence microscopy, and laser confocal imaging analysis

Cell transfections were performed using Lipofectamine 2000 (11668019; Thermo Scientific). An Olympus BX63 ﬂuorescence microscope or Olympus FV1200MPE two-photon laser confocal microscope was used to detect GFP-LC3 puncta, mitochondrial morphology, the colocalization of mitochondria and lysosome or the changes of red and green fluorescence of mito-dsRED2-EGFP. Cells were seeded into 35 mm glass bottom confocal dishes for confocal microscopy imaging. Mitochondria were stained with MitoTracker Red CMXRos (M7512; Invitrogen) or MitoTracker Green (C1048; Beyotime Biotechnology). Lysosomes were stained with Lysotracker Red (C1046; Beyo-time Biotechnology). Nuclei were stained with Hoechst Blue 33342 (C0031; Solarbio).

### Co-immunoprecipitation

Immunoprecipitation was performed using a commercially available kit named Pierce Classic Magnetic IP/Co-immunoprecipitation (Co-IP) Kit (88804; Thermo Scientific) according to the manufacturer’s protocol. Cells transfected with the appropriate plasmids were harvested and lysed with IP-lysis buffer (pH 7.4, 0.025 M Tris, 0.15 M NaCl, 0.001 M EDTA, 1% NP40, and 5% glycerol) with 1 mM PMSF and phosphatase inhibitors for 30 min at 4°C. Cell lysates were centrifuged at 12,000 rpm for 15 min at 4°C. The supernatant was collected, and protein concentrations were determined with a NanoDrop 2000 spectrophotometer. The protein extract was incubated with primary IP Abs overnight at 4℃ with rotation to form the immune complex. Then, the complex was added to the tube containing pre-washed magnetic beads and incubated at room temperature for 3 h with mixing. The magnetic beads were collected and washed three times. Proteins binding on the beads were eluted with elution buffer and boiled with loading buffer (1 M Tris-HCl, pH 6.8, 5% glycerol, 4% SDS, 0.02% bromophenol blue, and 10% beta-mercaptoethanol) for 10 min, and finally analyzed with western blot.

### Transmission electron microscopy

HEp-2 cells infected with RSV or transfected with HA-NS1 plasmid were harvested and fixed with 2.5% glutaraldehyde in 0.1 M sodium cacodylate buffer overnight at 4°C. Glutaraldehyde-fixed HEp-2 cells were postfixed with 1% osmic acid, dehydrated stepwise with ethanol, and embedded in epoxy resin. Ultrathin sections were cut using a Leica ultramicrotome and stained with uranyl acetate and lead citrate. The above-described procedure was done by the Electron Microscope Center of Hebei Medical College. Cells were imaged using a Hitachi 7500 transmission electron microscope at 80-kV acceleration voltage. Mitochondrial morphology and mitophagosomes were observed by transmission electron microscopy. Mitophagosomes were defined as double-membrane vacuoles containing normal or degenerating mitochondria with a diameter of 0.2–1.0 µm (58).

### Immunofluorescence analysis

HEp-2 cells were seeded on coverslips and transiently transfected with indicated plasmids with Lipofectamine 2000. Then, the cells were fixed with 4% formaldehyde for 15 min, followed by permeabilization with 0.5% Triton X-100 in PBS for 10 min at room temperature. The coverslips were blocked with 10% goat serum in PBST (blocking solution) for 1 h and incubated with primary antibodies diluted in blocking solution overnight at 4°C. Primary antibodies included mouse monoclonal anti-Flag (M185-3L; Medical & Biological Laboratories; 1:500) and rabbit polyclonal anti-TOM20 (11802-1-AP; Proteintech Group; 1:200). After three washes, cells were incubated with secondary antibodies for 1 h. Secondary antibodies contained Alexa 488-conjugated goat anti-mouse (ab150113; Abcam; 1:500) and Alexa 594-conjugated goat anti-rabbit (ab150080; Abcam; 1:500). Coverslips were mounted on the glass slides with DAPI Fluoromount-G (SouthernBiotech) and visualized by Olympus confocal microscope.

### Dual-Luciferase Reporter Assay

The plasmid for luciferase assays was a firefly luciferase reporter plasmid containing *IFNB1* promoter (pGL4-IFNβ-luc). In brief, a 1,919-bp promoter of the *IFNB1* gene, corresponding to the sequence from nt −1862 to +57 (relative to the transcriptional start site) of the 5′-flanking region of the *IFNB1* gene, was generated from human genomic DNA by PCR using forward 5′-ctaGCTAGC**CTCCAAGTGCACGAAATTA**-3′, and reverse 5′-cccAAGCTT**GTGTCGCC TACTACCTGTT**-3′ primers. The PCR product was gel purified and cloned into the NheI and HindIII sites of the pGL4.10-Basic vector. pRL-TK *Renilla* luciferase plasmid (for normalization of transfection efficiency) was obtained from Invitrogen. HEK 293 cells were seeded in six-well plates and co-transfected with 1 µg/well of pGL4-IFNβ-luc, 0.1 µg/well of pGL4-TK, 1 µg/well of RIGI-CARD-expressing plasmid (RIGI, preserved in our lab), 1.5 µg/well of NS1 expressing plasmid, and 1 µg/well of TUFM expressing plasmid or empty vectors in control or TUFM knockdown cells with siRNA (co-transfected with pGL4-IFNβ-luc, pRL-TK plasmid, empty vectors, and siNC as a control group). Cells were lysed with passive lysis buffer with gentle shaking at 30 h post-transfection. Subsequently, the *Renilla* and firefly luciferase activities were measured by Dual-Luciferase Reporter Assay System (E2920, Promega) according to the manufacturer’s protocol, and luminescence was measured with BioTek Synergy HT2 multiscan spectrum. Relative ratio = (well A1 *firefly* luminescence/*Renilla* luminescence)/(control *firefly* luminescence/*Renilla* luminescence).

### RNA extraction and quantitative real-time PCR analysis

Total cellular RNA was extracted with the TRIzol reagent (DP424; TIANGEN) according to the manufacturer’s protocol. The RNA was reverse transcribed to cDNA using the PrimeScript RT reagent kit with genomic DNA (gDNA) Eraser (RR047A; TaKaRa). qRT-PCR was performed with cDNA templates and the PowerUp SYBR green master mix (A25741; Applied Biosystems) and analyzed using the ABI prism 7500 Sequence detection system (Applied Biosystems). The relative expressions of target mRNA were normalized to the expression of β-actin using the 2^−ΔΔ*C*t^ method (where *C*_*t*_ is the threshold cycle). The primers were shown in Table 2 and synthesized by Sangon Biotech.

**TABLE 2:** List of primers used in the study

Genes	Sequence	
RSV NS1	FORWARD	5′-CACAACAATGCCAGTGCTACAA-3′
RSV NS1	REVERSE	5′-TTAGACCATTAGGTTGAGAGCAATGT-3′
RSV N	FORWARD	5′-AAGGGATTTTTGCAGGATTGTTT-3′
RSV N	REVERSE	5′-CTCCCCACCGTAGCATTACTTG-3′
RSV M	FORWARD	5′-ATGTGCTAATGTGTCCTTGGATGA-3′
RSV M	REVERSE	5′-TGATTTCACAGGGTGTGGTTACA-3′
RSV G	FORWARD	5′-CGGCAAACCACAAAGTCACA-3′
RSV G	REVERSE	5′-TTCTTGATCTGGCTTGTTGCA-3′
RSV M2	FORWARD	5′-CATGAGCAAACTCCTCACTGAACT-3′
RSV M2	REVERSE	5′-TCTTGGGTGAATTTAGCTCTTCATT-3′
RSV L	FORWARD	5′-CACTCTACAAAACAAAAAGACACAATCA-3′
RSV L	REVERSE	5′-AGGATGCTGCATTGAACACATT-3′
RSV NS2	FORWARD	5′-ATGGACACAACCCACAATGATACCAC-3′
RSV NS2	REVERSE	5′-ATTGTAGTCTCAAGTGACAACGGTCTC-3′
RSV P	FORWARD	5′-AGTGCAGGACCTACATCTGCTC-3′
RSV P	REVERSE	5′-AGCTGTTGGCTATGTCCTTGG-3′
IFNβ-Human	FORWARD	5′-GCTTGGATTCCTACAAAGAAGCA-3′
IFNβ-Human	REVERSE	5′-ATAGATGGTCAATGCGGCGTC-3′
TUFM-Human	FORWARD	5′-GGGGCTAAGTTCAAGAAGTACG-3′
TUFM-Human	REVERSE	5′-CACATGAGCCGCATTGATGG-3′
β-actin-Human	FORWARD	5′-CCTGGCACCCAGCACAAT-3′
β-actin-Human	REVERSE	5′-GGGCCGGACTCGTCATAC-3′
IFNβ-Mouse	FORWARD	5′-AGCTCCAAGAAAGGACGAACA-3′
IFNβ-Mouse	REVERSE	5′-GCCCTGTAGGTGAGGTTGAT-3′
TUFM-Mouse	FORWARD	5′-GACAAGCCCCATGTGAATGTG-3′
TUFM-Mouse	REVERSE	5′-CCCTCGGCTAGAATTTTCGTGA-3′
β-actin-Mouse	FORWARD	5′-CTACCTCATGAAGATCCTGACC-3′
β-actin-Mouse	REVERSE	5′-CACAGCTTCTCTTTGATGTCAC-3′

### Detection of relative mtDNA content

Total DNA was isolated from the cells using WizardSV Genomic DNA Purification System (A2360; Promega). The DNA concentration was measured by NanoDrop 2000c Spectrophotometer. Relative mtDNA content was measured by qRT-PCR using 2^−ΔΔCt^ method and expressed as mtDNA/nDNA ratios, as described in previous studies (59). The reference single-copy nuclear gene (nDNA) used for normalization in this study was human *beta-globin* (*HBB*). The primers for the mitochondrial DNA and *HBB* were previously described (60) and as follows.

Mt forward: 5′-CACCCAAGAACAGGGTTTGT-3′,

Mt reverse: 5′-TGGCCATGGGTATGTTGTTA-3′;

*HBB* forward: 5′-GCTTCTGACACAACTGTGTTCACTAGC-3′

*HBB* reverse: 5′-CACCAACTTCATCCACGTTCACC-3′.

### CRISPR/Cas9-mediated gene knockout or knockdown

HEp-2 cells stably expressing specific sgRNAs against *TUFM*, *TAX1BP1*, *SQSTM1*, *OPTN*, *CALCOCO2, NBR1,* and *DNM1L* were established by being transduced with lentiviral particles expressing specific sgRNA targeting *DNM1L* or transfected with px459 plasmid containing sgRNA sequence targeting *TUFM*, *TAX1BP1*, *SQSTM1*, *OPTN*, *CALCOCO2,* or *NBR1*. Lentiviral particles were packaged by transfection HEK 293T cells with YKO-sgRNA Lentivectors (Ubigene Biosciences), psPAX2, and pMD2.G at a ratio of 4:3:1, respectively. Viral supernatants were collected at 48 h post-transfection. After infection with lentiviral particles or transfection, HEp-2 cells were treated with hygromycin (200 µg/mL, BIOFROXX) or puromycin (3 µg/mL, Biosharp) for selection. Surviving cells were used as knockdown cells, and the knockdown efficiency was determined by western blotting. To generate TUFM KO HEp-2 cells, surviving cells were further reseeded as single colonies in 96-well plates. After 2–3 weeks, stable TUFM KO cell clones were screened based on western blotting. In addition, genomic DNA was extracted from the cell lines from single clones, and PCR analysis was performed to amplify targeted loci. Agarose gel electrophoresis was used to confirm the correct size of PCR products. PCR products were then cloned into the ZTOPO-Blunt/TA vector and transformed into DH5a-competent cells. Plasmid DNA was isolated from multiple colonies of each transformation and sequenced to ensure frameshift mutations in the targeted region. The sgRNA sequences were as follows:

*TUFM* sgRNA: GATGGTACCCACATTCACATG **TGG**;

*TAX1BP1* sgRNA: CAGGTGTGCATTAGGA **AGG**;

*SQSTM1* sgRNA: CGGCTCAGCAGCCGCTCGCA **GGG**;

*OPTN* sgRNA: CCTCCGGGGTAAACGTGTCC **AGG;**

*CALCOCO2* sgRNA: GTGAGTATTACACCTTCATG **TGG;**

*NBR1* sgRNA: CTGCAGATGCAAGTC CACGA **AGG;**

*DNM1L* sgRNA1: GGTGACAATTCCAGTACCTC **TGG;**

The bold nucleotides are a protospacer-adjacent motif sequence not included in the sgRNA but recognized by the Cas9 protein.

### siRNA knockdown

For the RNA interference knockdown experiments, small interfering RNA (siRNA) against human *TUFM* and *PARK2* were designed and synthesized from GenePharma. Cells were transiently transfected with 30 nM siRNA using Lipofectamine 2000 according to the manufacturer’s protocols. At 12 h post-transfection with siRNA, the cells were further infected with RSV or transfected with indicated plasmids. A scrambled siRNA was used as a negative control. The silencing efficiency was determined by western blotting. The sequences of siRNA duplex used were as follows:

*TUFM* siRNA (sense): 5′-GGGAGCUGCUCACCGAGUUTT-3′,

*TUFM* siRNA (antisense): 5′-AACUCGGUGAGCAGCUCCCTT-3′;

*PARK2* siRNA (sense): 5′-GCCACGUGAUUUGCUUAGATT-3′,

*PARK2* siRNA (antisense): 5′-UCUAAGCAAAUCACGUGGCTT-3′;

scrambled siRNA (sense): 5′-UUCUCCGAACGUGUCACGUTT-3′,

scrambled siRNA (antisense): 5′-ACGUGACACGUUCGGAGAATT-3′.

### Biochemical intervention

For detection of autophagic flux, HEp-2 cells were pretreated with 10 nM bafilomycin A1 for 2 h, followed by RSV infection or transfection with corresponding plasmids for 30 h in the presence of bafilomycin A1. As a positive control of mitochondria fission and mitophagy, the cells were treated with 20 µM CCCP for 6 h. To inhibit DRP1 activity and mitochondria fission, the cells were pretreated with 10 µM Mdivi-1 for 2 h and followed by RSV infection or transfection with corresponding plasmids in the presence of Mdivi-1.

### Extraction of mitochondrial and cytoplasmic protein

Mitochondria fractions were isolated with the Mitochondria Isolation Kit for Mammalian Cells (C0010; Applygen Technology) according to the protocol. In brief, 2 × 10^7^ cells were collected and suspended in 1.5 mL of pre-cooled Mito Solution. Homogenize cells with Dounce homogenizer on ice to effectively lyse the cells (about 30–50 strokes). Then, the cell homogenate was centrifuged at 800 × *g* for 5 min at 4°C, and the supernatant was collected. Repeat this step once, and the supernatant was centrifuged at 10,000 × *g* for 10 min at 4°C. Finally, the supernatant was collected as cytoplasmic proteins. The remaining mitochondria pellets were washed with 0.2 mL Mito Solution and collected by centrifuging at 12,000 × *g* for 10 min at 4°C. Approximately 100 µL of RIPA was added to the mitochondria pellets, and the mixture was placed on ice for 30 min and then centrifuged at 13,000 rpm for 10 min at 4°C. The supernatant was collected as mitochondrial proteins. Cytoplasmic and mitochondrial protein concentration was determined by NanoDrop 2000c spectrophotometer.

### Mitochondrial potential measurement by JC-1 staining and fluorescence microscope detection

HEp-2 cells were stained with a Mitochondrial membrane potential JC-1 assay kit (M8650; Solarbio) according to the manufacturer’s protocols. Approximately 1 × 10^6^ HEp-2 cells were plated in six-well plates and infected with RSV or mock-infected with PBS for 24 h. After infection, the cells were washed and incubated with 1 mL of 1 × JC-1 working solution at 37°C, 5% CO_2_ for 20 min, washed three times with 1 mL of 1 × JC-1 buffer, then analyzed by fluorescence microscope to observe the fluorescence intensity of JC-1 monomer and aggregates. JC-1 dye forms red-fluorescent aggregates in healthy cells with a high mitochondrial ΔΨm, while it exhibits a green-fluorescent monomer at low membrane potential. For the positive control tube, cells were treated with 20 µM CCCP supplied with the kit at 37°C for 20 min.

### Expression and purification of the His-TUFM and His-NS1 protein

Homo TUFM and RSV NS1 coding region was amplified from pCMV3-N-HA-TUFM (HG16801-NY; Sino Biological) and pCAGGS-Flag-NS1 plasmid containing the codon-optimized RSV NS1 gene (produced by *GenScript*) by PCR and was cloned into prokaryotic expression vector pET-28a^+^. The correctly sequenced recombinant plasmid was transformed into *E. coli BL21 (DE3*) competent cells. The expression of recombinant plasmid was induced in *E. coli BL21* by IPTG (1.0 mM). Fusion proteins His-TUFM and His-NS1 were purified by Ni-NTA beads and verified by SDS-PAGE and Western blot.

### H&E staining

The histology of the RSV-infected mouse lung tissue was analyzed using H&E staining. In brief, the mouse lungs were fixed with 4% polyformaldehyde, dehydrated through a graded alcohol series, embedded in paraffin, cut into 5-µm-thick sections, and stained with H&E. The slides were examined by light microscopy. H＆E slides were scored blindly according to the following criteria: 0 = no lesions; 1 = focal to few multifocal mild lesions; 2 = multiple multifocal mild-to-moderate lesions; 3 = multifocal areas of moderate-to-severe lesions; and 4 = extensive areas of lesions and necrosis.

### ELISA

IFNβ ELISA kit (E-NW0026Hu; ENOVA, China) was used to determine the concentration of IFNβ. In 96-well plates, standards (50 µL) and samples (10 µL serum and 40 µL diluent) were added into predefined wells, while blank wells were left empty. In the wells for standards and samples, horseradish peroxidase-labeled conjugates (100 µL) were added before sealing the plates for incubation at 37°C for 2 h. After washing the plates five times, substrates A (50 µL) and B (50 µL) were added to each well. After incubation at 37°C for 15 min, stop solution (50 µL) was added into each well, and absorbance of each well was measured at 450 nm within 15 min.

### Statistical analysis

Data were expressed as means ± standard deviations. All experiments were performed in triplicate or more replicates. SPSS statistical software (version 16.0) was used for statistical analyses. The significance between the two groups was determined using Student’s *t* test. Statistical analyses for multiple groups were performed using one-way analysis of variance. The Mann-Whitney rank-sum test was applied for non-normally distributed data. A *P* value of <0.05 was considered significant statistically.

## SUPPLEMENTAL MATERIAL

Fig. S1 mbio.01480-23-s0001.tif RSV infection and RSV-NS1 protein could induce complete mitophagy.

Fig. S2 mbio.01480-23-s0002.tif RSV infection and RSV-NS1 protein could induce mitophagy by facilitating Drp1-dependent mitochondria fission.

Fig. S3 mbio.01480-23-s0003.tif Inhibition of autophagy significantly blocked the inhibitory effect of RSV-NS1 on the IFNβ level driven by RIG1 or RSV infection.

Fig. S4 mbio.01480-23-s0004.tif The colocalization of Flag-NS1 and TOMM20 depends on TUFM.

Fig. S5 mbio.01480-23-s0005.tif RSV/RSV-NS1 protein mediates TUFM-dependent pro-viral autophagy.

Fig. S6 mbio.01480-23-s0006.tif RSV-NS1 could induce mitophagy independent on known mitophagy receptors, and its LIR motif is essential for interaction with LC3B and inhibitory effect of NS1 on IFNβ production.

Fig. S7 mbio.01480-23-s0007.tif TUFM could suppress relative expression levels of RSV genes in vivo.

Fig. S8 mbio.01480-23-s0008.tif The co-localization of Flag-NS1 and TUFM in vivo.

Supplemental legends mbio.01480-23-s0009.docx Legends for Fig. S1 to S8.

ASM does not own the copyrights to Supplemental Material that may be linked to, or accessed through, an article. The authors have granted ASM a non-exclusive, world-wide license to publish the Supplemental Material files. Please contact the corresponding author directly for reuse.
