# Supplementary figures and images for: The nonstructural protein 1 of respiratory syncytial virus hijacks host mitophagy as a novel mitophagy receptor to evade the type I IFN response in HEp-2 cells
Source: mBio. 2023 Nov 1;14(6):e01480-23. doi: 10.1128/mbio.01480-23 (PMC10746179; doi:10.1128/mbio.01480-23)

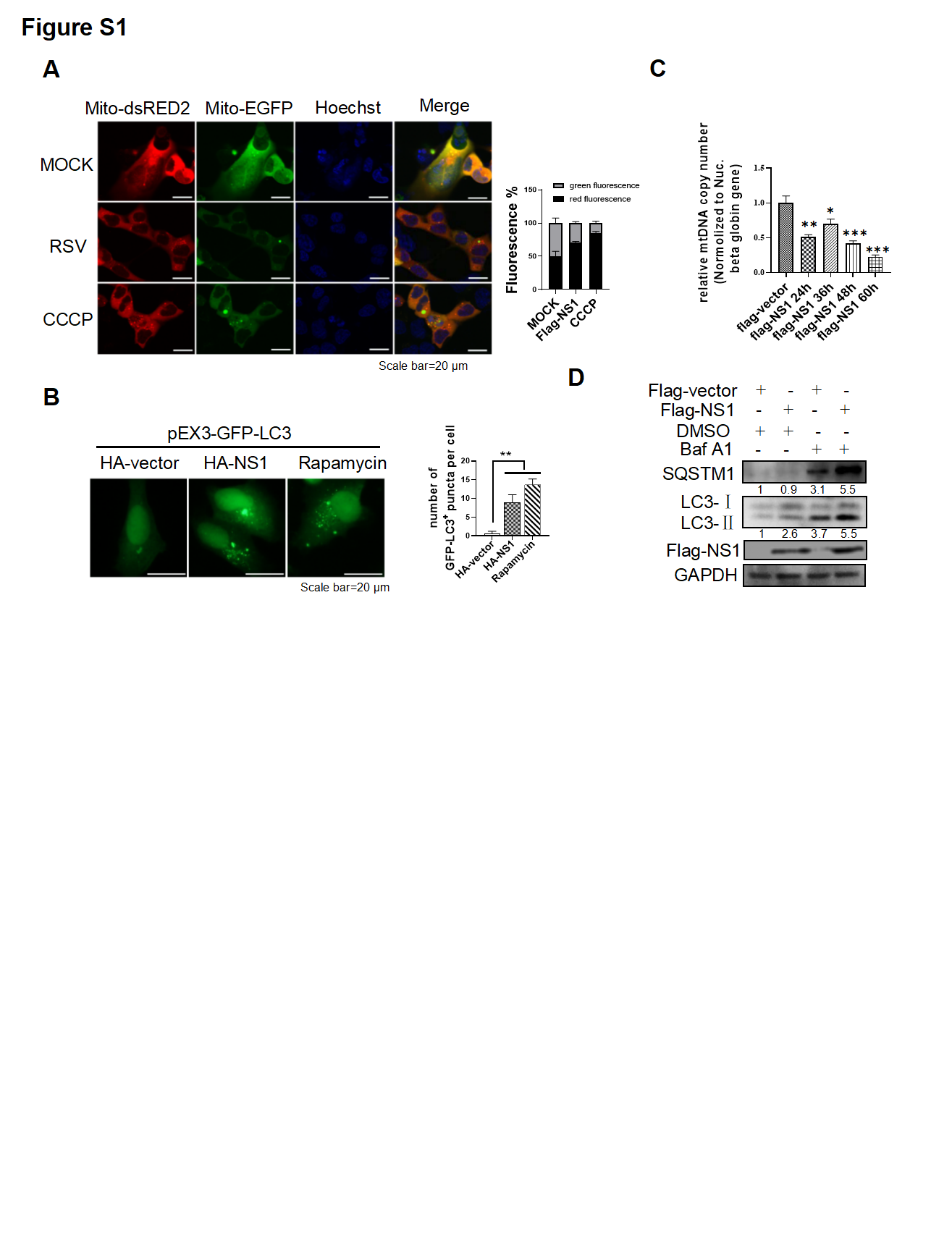

Supplement: Fig. S1 — RSV infection and RSV-NS1 protein could induce complete mitophagy. [file mbio.01480-23-s0001.tif]

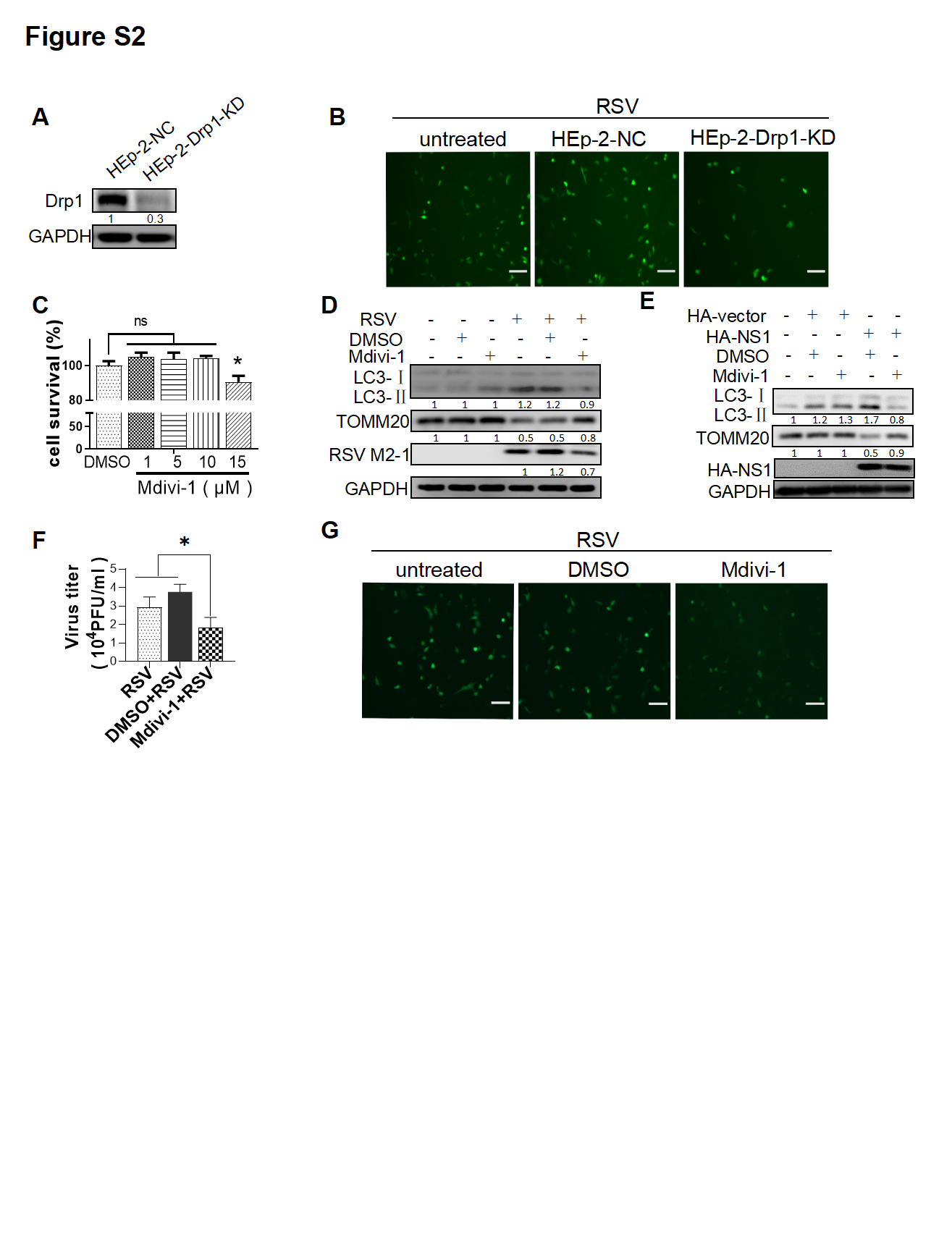

Supplement: Fig. S2 — RSV infection and RSV-NS1 protein could induce mitophagy by facilitating Drp1-dependent mitochondria fission. [file mbio.01480-23-s0002.tif]

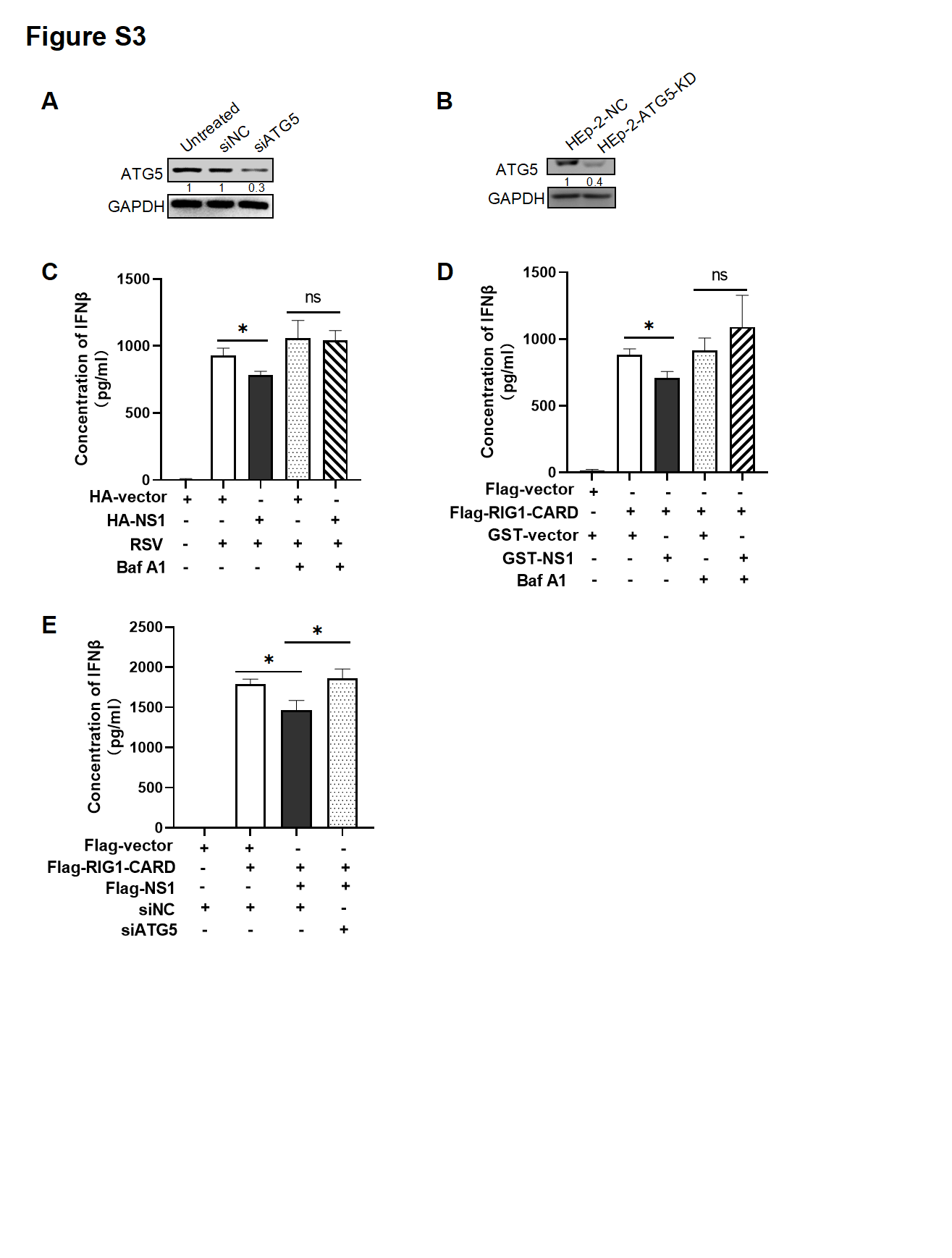

Supplement: Fig. S3 — Inhibition of autophagy significantly blocked the inhibitory effect of RSV-NS1 on the IFNβ level driven by RIG1 or RSV infection. [file mbio.01480-23-s0003.tif]

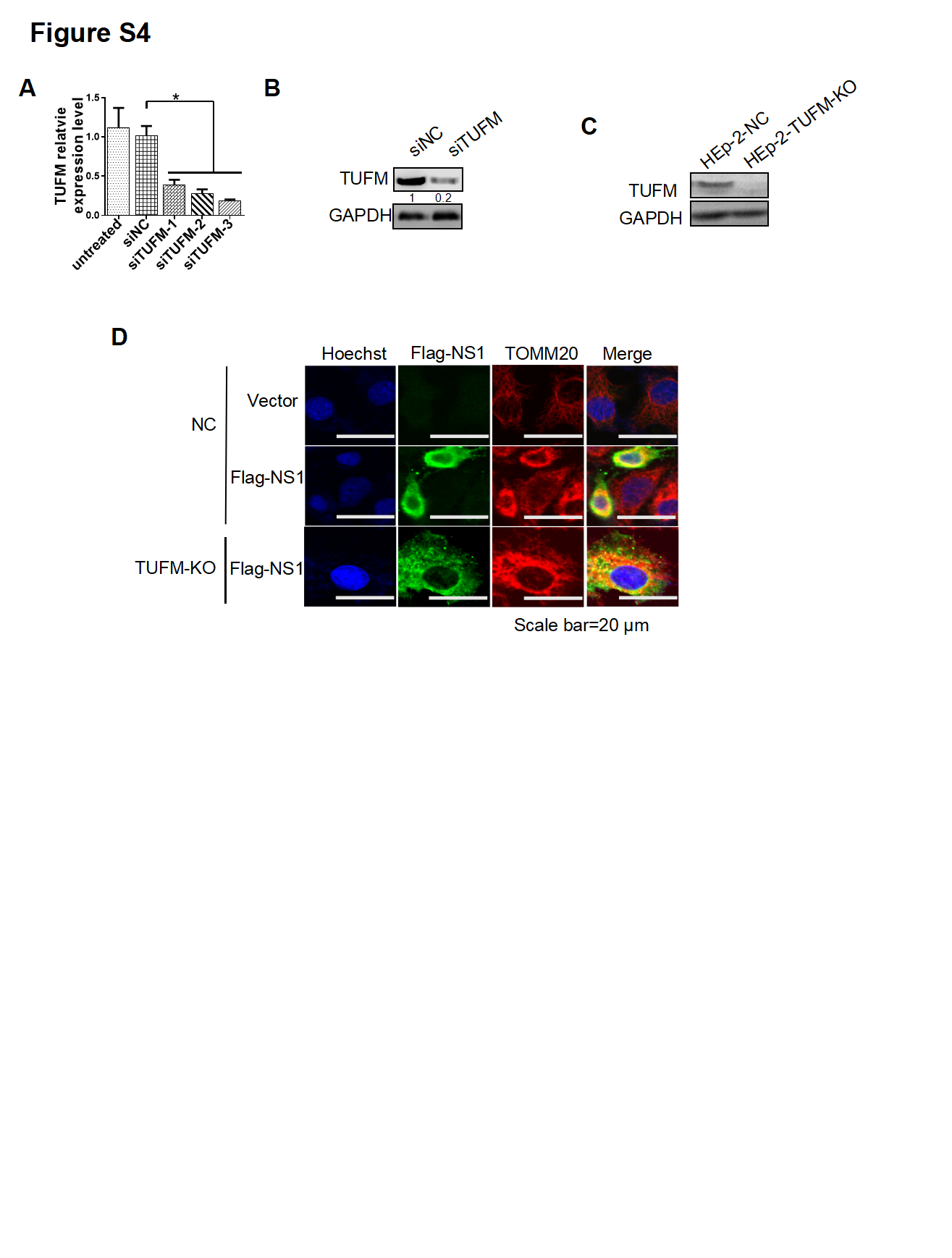

Supplement: Fig. S4 — The colocalization of Flag-NS1 and TOMM20 depends on TUFM. [file mbio.01480-23-s0004.tif]

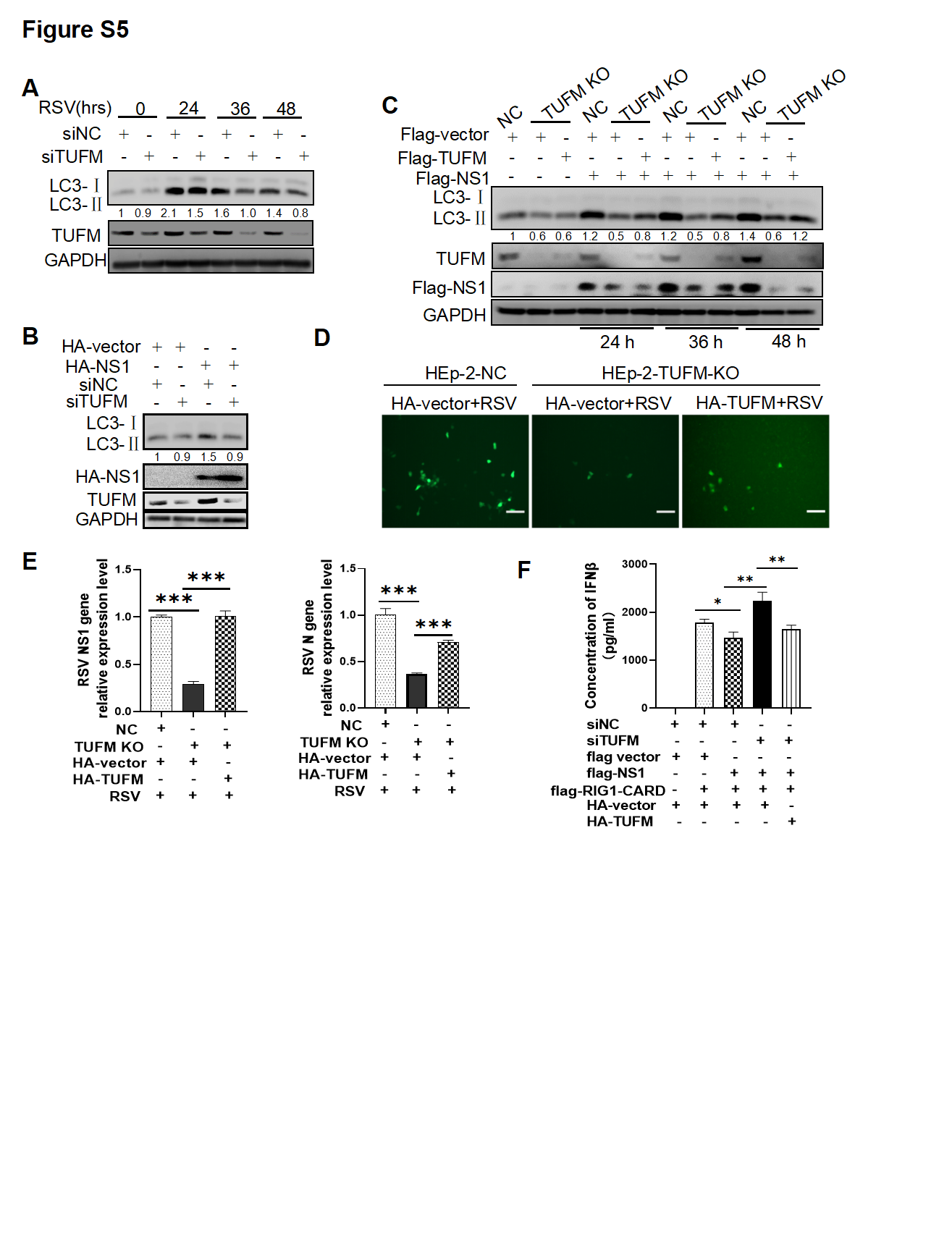

Supplement: Fig. S5 — RSV/RSV-NS1 protein mediates TUFM-dependent pro-viral autophagy. [file mbio.01480-23-s0005.tif]

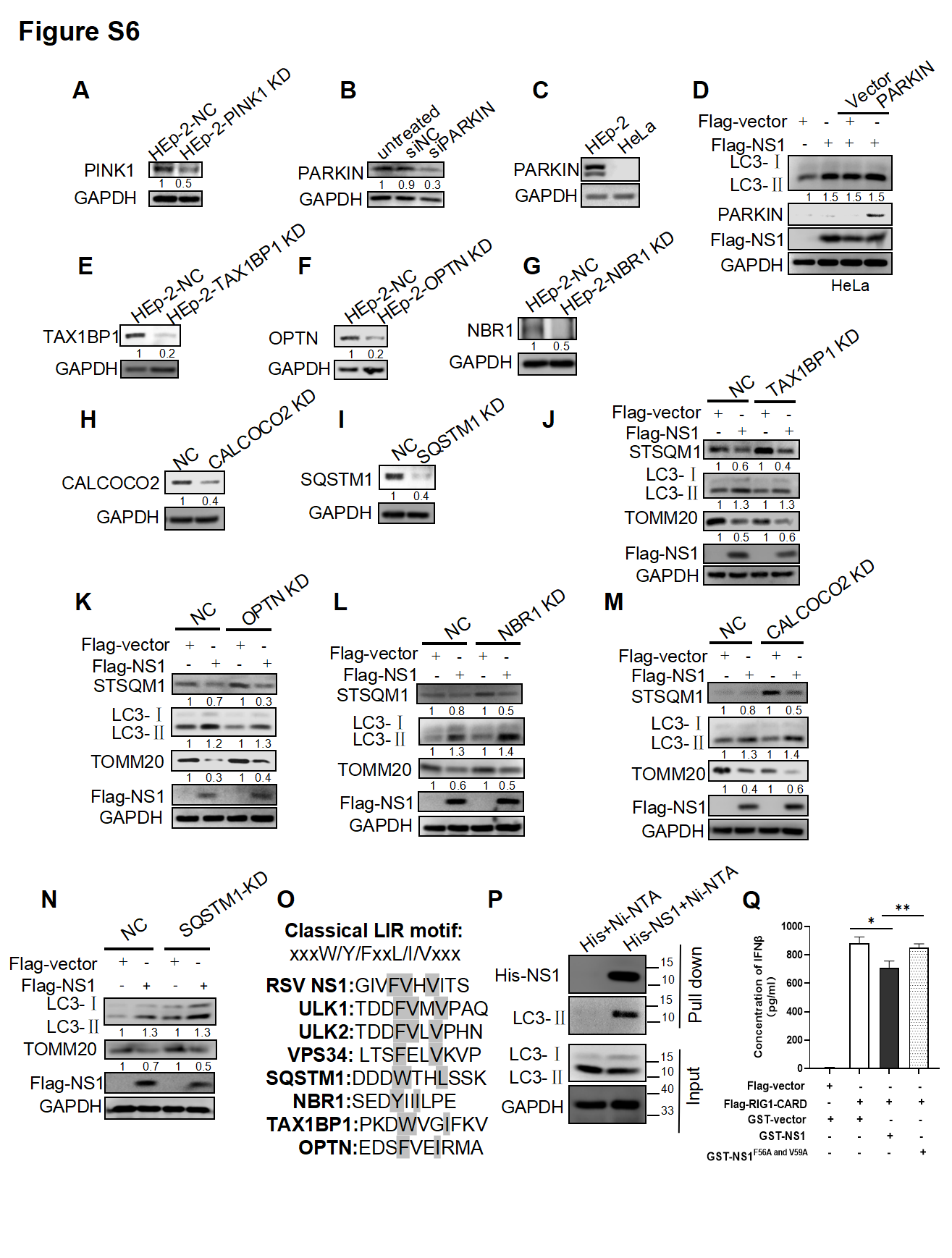

Supplement: Fig. S6 — RSV-NS1 could induce mitophagy independent on known mitophagy receptors, and its LIR motif is essential for interaction with LC3B and inhibitory effect of NS1 on IFNβ production. [file mbio.01480-23-s0006.tif]

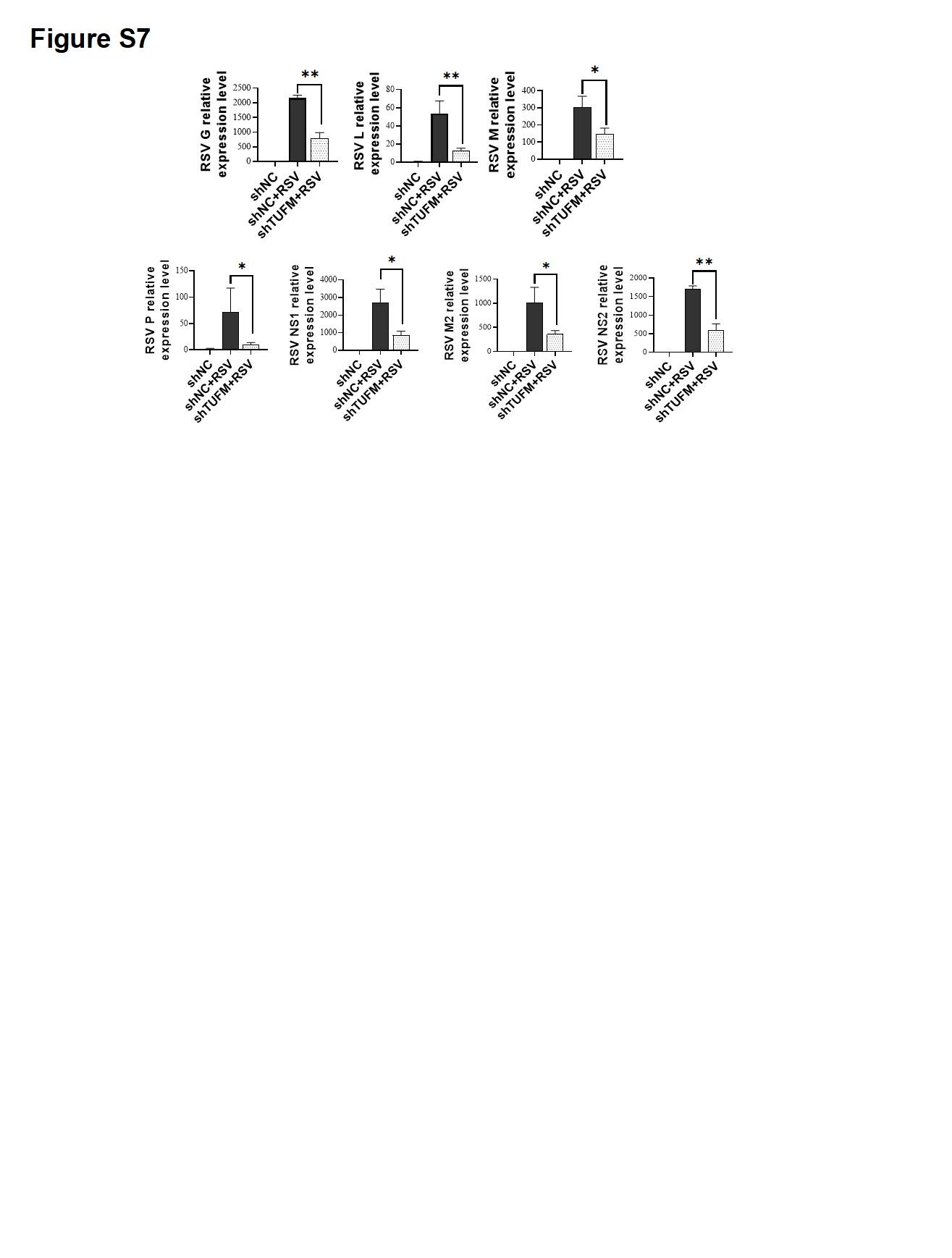

Supplement: Fig. S7 — TUFM could suppress relative expression levels of RSV genes in vivo. [file mbio.01480-23-s0007.tif]

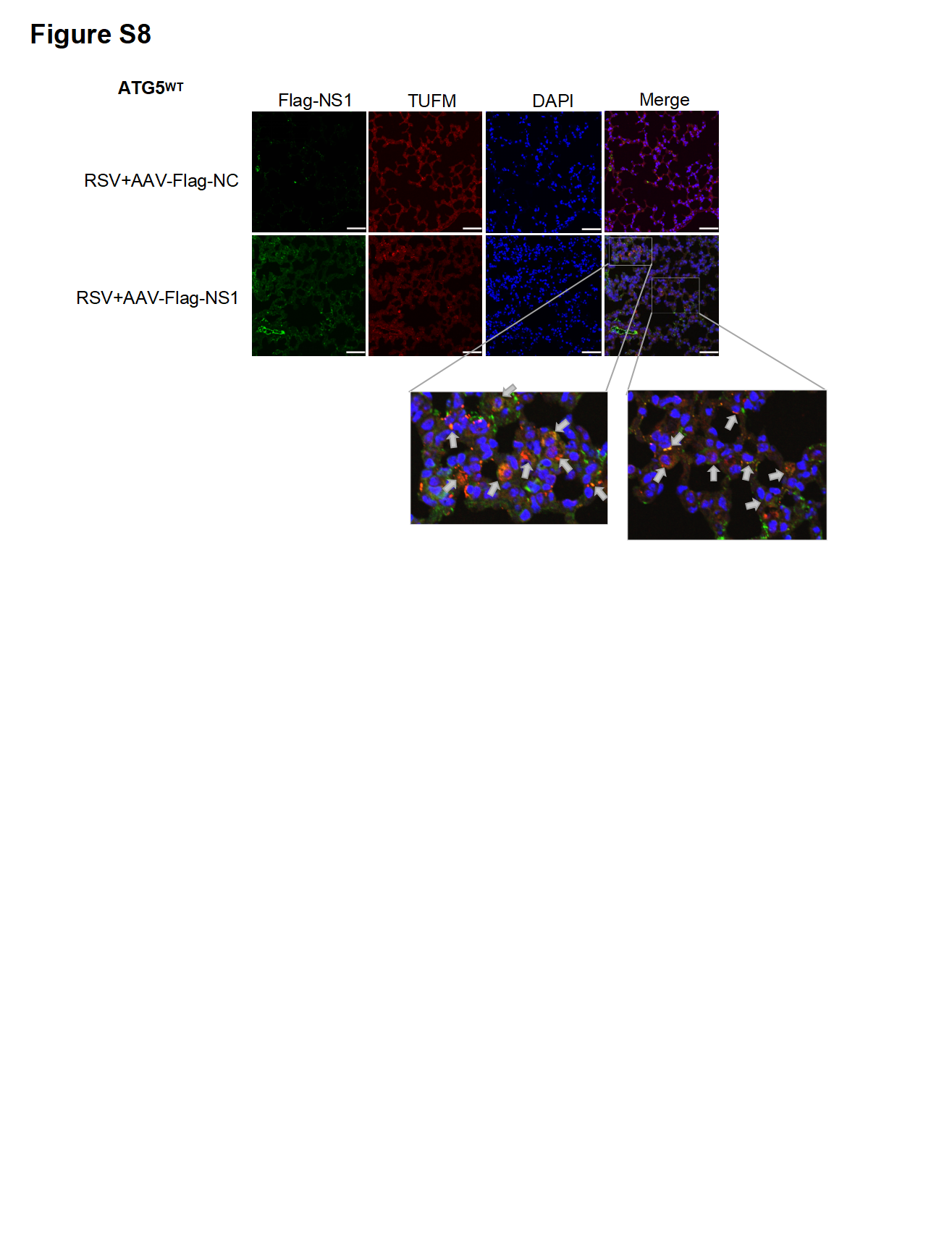

Supplement: Fig. S8 — The co-localization of Flag-NS1 and TUFM in vivo. [file mbio.01480-23-s0008.tif]
