# Supplementary material for: The nonstructural protein 1 of respiratory syncytial virus hijacks host mitophagy as a novel mitophagy receptor to evade the type I IFN response in HEp-2 cells
Source: mBio. 2023 Nov 1;14(6):e01480-23. doi: 10.1128/mbio.01480-23 (PMC10746179; doi:10.1128/mbio.01480-23)
Supplement: Supplemental legends — Legends for Fig. S1 to S8. [file mbio.01480-23-s0009.docx]

**Supplemental Figure legends**

**Figure S1. RSV infection and RSV-NS1 protein could induce complete mitophagy.**

(A) HEK 293 cells were transiently transfected with mito-dsRED2-EGFP plasmid for 24 h, followed by RSV infection for another 24 h. The changes of red and green fluorescence were observed by a laser confocal microscope to reflect the delivery of mitochondria to lysosomes during mitophagy. CCCP (20 µM) was used as a positive control to induce mitophagy. Scale bar = 20 µm.

(B) HEp-2 cells were transfected with pEX3-GFP-LC3 plasmids for 24 h, followed by transfection with pCAGGS-HA or pCAGGS-HA-NS1 for another 24 h, and then analyzed by fluorescence microscopy for the presence of fluorescent GFP-LC3 puncta. Rapamycin was used as a positive control. The bar graph represents the number of puncta per GFP-LC3 positive cell. The data are from 50 cells per sample. Scale bar = 20 μm.

(C) HEp-2 cells were transfected with pCAGGS-Flag or pCAGGS-Flag-NS1 plasmids for indicated times. DNAs were isolated from cells, and the mitochondrial DNA (mtDNA) content normalized to single-copy nuclear gene (nDNA) HBB (mtDNA/nDNA ratios). Each data represents the mean±SD of 3 independent experiments.

(D) HEp-2 cells were transfected with pCAGGS-Flag or pCAGGS-Flag-NS1 plasmids for 36 h in the absence or presence of Baf A1 (10 nM). Cell lysates were analyzed by WB.

Each data represents the mean±SD of 3 independent experiments. *: *P*＜0.05; **: *P*＜0.01; ***: *P*＜0.001.

**Figure S2. RSV infection and RSV-NS1 protein could induce mitophagy by facilitating Drp1-dependent mitochondria fission.**

(A) Drp1 knockout HEp-2 cells (HEp-2-Drp1-KD) were generated using the CRISPR/Cas9 system. And the knockout efficiency was validated by WB.

(B) HEp-2 cells, HEp-2-NC cells and HEp-2-Drp1-KD cells were infected with RSV for 24 h. The GFP fluorescence of GFP-RSV were detected by fluorescence microscopy. Scale bar = 27 mm.

(C) HEp-2 cells were treated with different concentrations of Mdivi-1, and cell viability was determined by CCK8 assay.

(D and E) HEp-2 cells were mock infected or infected with RSV (D) for 24 h or transfected with pCAGGS-HA or pCAGGS-HA-NS1 plasmids (E) for 36 h in the absence or presence of Mdivi-1 (10 µM). The expression of LC3-Ⅱ, TOMM20, and viral proteins were analyzed by WB.

(F and G) HEp-2 cells were infected with RSV in the absence or presence of Mdivi-1 (10 µM) for 24 h. The GFP fluorescence of GFP-RSV and total viral titers were detected by viral plaque assay (F) and fluorescence microscopy (G). Scale bar = 27 mm.

Each data represents the mean±SD of 3 independent experiments. *: *P*＜0.05; **: *P*＜0.01; ***: *P*＜0.001.

**Figure S3. Inhibition of autophagy significantly blocked the inhibitory effect of RSV-NS1 on the IFNβ level driven by RIG1 or RSV infection.**

(A) HEK 293 cells were transfected with siNC or siATG5 for 24 h. The expression of ATG5 proteins was detected by WB.

(B) ATG5 knockdown HEp-2 cells (HEp-2-ATG5-KD) were generated using the CRISPR/Cas9 system. And the knockdown efficiency was validated by WB.

(C, D and E) HEK 293 cells were treated with Baf A1 (C and D) or transfected with siATG5 (E), and then were co-transfected with indicated plasmids with (C) or without (D and E) RSV-infection. The level of IFNβ in cell lysates was measured by ELISA.

Each data represents the mean±SD of 3 independent experiments. *: *P*＜0.05; **: *P*＜0.01; ***: *P*＜0.001.

**Figure S4. The colocalization of Flag-NS1 and TOMM20 depends on TUFM.**

(A and B) HEp-2 cells were transfected with siNC or three different siTUFM (siTUFM-1, siTUFM-2, siTUFM-3) for 24 h. TUFM mRNA relative expression levels were detected by qRT-PCR (A), and the expression of TUFM proteins was detected by WB (B).

(C) Single cell-derived TUFM knockout HEp-2 cells (HEp-2-TUFM-KO) were generated using the CRISPR/Cas9 system. And the knockout efficiency was validated by WB.

(D) Negative Control (NC)- or HEp-2-TUFM KO cells were transfected with pCAGGS-Flag or pCAGGS-Flag-NS1 plasmids for 24 h, and then were immuno-labeled with anti-flag and anti-TOMM20. A laser confocal microscope observed the colocalization of Flag-NS1 and TOMM20. Nuclei were stained with Hoechst. Scale bar=20 µm.

Each data represents the mean±SD of 3 independent experiments. *: *P*＜0.05; **: *P*＜0.01; ***: *P*＜0.001.

**Figure S5. RSV/RSV-NS1 protein mediates TUFM-dependent pro-viral autophagy.**

(A) HEp-2 cells transfected with siNC or siTUFM were infected with RSV for indicated times. The expression of LC3-Ⅱ and TUFM proteins were analyzed by WB.

(B) HEp-2 cells were transfected with siNC or siTUFM for 16 h and followed by transfection with pCAGGS-HA or pCAGGS-HA-NS1 for 36 h. The expression of LC3-Ⅱ, HA-NS1, and TUFM proteins were detected by WB.

(C) HEp-2-NC or HEp-2-TUFM-KO cells were co-transfected with pCAGGS-Flag/pCAGGS-Flag-TUFM and pCAGGS-Flag-NS1 plasmids as indicated. The expression of LC3-Ⅱ, TUFM, and Flag-NS1 proteins were detected by WB.

(D and E) HEp-2-NC, HEp-2-TUFM-KO cells, or HEp2-TUFM-KO cells transfected with control or TUFM plasmids were infected with RSV for 24 h. The GFP fluorescence of GFP-RSV (D), viral N and NS1 gene expression (E) were detected by RT-qPCR and fluorescence microscopy. Scale bar=27 mm.

(F) HEK 293 cells were transfected with siNC or siTUFM for 16 h, then were transfected with indicated plasmids for 36 h. The level of IFNβ in cell lysates was measured by ELISA.

Each data represents the mean±SD of 3 independent experiments. *: *P*＜0.05; **: *P*＜0.01; ***: *P*＜0.001.

**Figure S6. RSV-NS1 could induce mitophagy independent on known mitophagy receptors and its LIR motif is essential for interaction with LC3B and inhibitory effect of NS1 on IFNβ production.**

(A) PINK1 knockdown HEp-2 cells (HEp-2-PINK1-KD) were generated using the CRISPR/Cas9 system. And the knockdown efficiency was validated by WB.

(B) HEp-2 cells were transfected with siNC or siPARKIN for 24 h. The knockdown efficiency of PARKIN was confirmed by WB.

(C and D) The expression level of PARKIN protein in HEp-2 and HeLa cells was determined by WB. HeLa cells were co-transfected with pCDH/pCDH-Parkin and pCAGGS-Flag-NS1 plasmids for 36 h. The expression level of LC3-Ⅱ, PARKIN, and Flag-NS1 proteins were detected by WB.

(E-N) Stable NBR1/TAX1BP1/SQSTM1/OPTN/CALCOCO2 knockdown HEp-2 cells (E-I) was generated by using the CRISPR/Cas9 system. HEp-2-TAX1BP1-KD (J), HEp-2-OPTN-KD (K), HEp-2-NBR1-KD (L), HEp-2-CALCOCO2-KD (M), HEp-2-SQSTM1-KD (N) and HEp-2-NC cells were transfected with a pCAGGS-Flag-NS1 plasmid for 36 h, and the expression of indicated proteins were detected by WB.)

(O) Classical LIR motif is presented in RSV NS1 protein and basal or core autophagy machinery proteins.

(P) Purified His or His-NS1 protein was incubated with lysates of HEK 293 cells and then subjected to Ni-NTA pulldown followed by immunoblots with anti-His and anti-LC3 antibodies.

(Q) HEK 293 cells were co-transfected with indicated plasmids for 36 h. The level of IFNβ in cell lysates was measured by ELISA.

Each data represents the mean±SD of 3 independent experiments. *: *P*＜0.05; **: *P*＜0.01; ***: *P*＜0.001.

**Figure S7. TUFM could suppress relative expression levels of RSV genes in vivo.**

Wide-type control and TUFM knockdown Balb/c mice were generated and then infected with RSV according to the protocols described in Materials and Methods. The relative expression levels of RSV genes of mouse lungs were detected by qRT-PCR. Gene expression was calculated by comparison to that of negative control mice. Results are means±SD for 5 mice per group.

Results are means±SD for 5 mice per group. *: *P*＜0.05; **: *P*＜0.01, ***: *P*＜0.001.

**Figure S8. The co-localization of Flag-NS1 and TUFM *in vivo.***

ATG5^WT^ C57BL/6 mice were infected with AAV-flag-NC or AAV-flag-NS1 followed by infection with RSV. Mouse lungs were harvested and immuno-labeled with anti-flag and anti-TUFM. The nucleus was stained with DAPI. Sample were visualized by laser confocal microscope. Scale bar=50 µm.
